# Follicle-stimulating hormone orchestrates glucose-stimulated insulin secretion of pancreatic islets

**DOI:** 10.1038/s41467-023-42801-6

**Published:** 2023-11-01

**Authors:** Yi Cheng, Hong Zhu, Jun Ren, Hai-Yan Wu, Jia-En Yu, Lu-Yang Jin, Hai-Yan Pang, Hai-Tao Pan, Si-Si Luo, Jing Yan, Kai-Xuan Dong, Long-Yun Ye, Cheng-Liang Zhou, Jie-Xue Pan, Zhuo-Xian Meng, Ting Yu, Li Jin, Xian-Hua Lin, Yan-Ting Wu, Hong-Bo Yang, Xin-Mei Liu, Jian-Zhong Sheng, Guo-Lian Ding, He-Feng Huang

**Affiliations:** 1 Obstetrics and Gynecology Hospital, Institute of Reproduction and Development, Fudan University, Shanghai, China; 2 Research Units of Embryo Original Diseases, Chinese Academy of Medical Sciences, Shanghai, China; 3 Key Laboratory of Reproductive Genetics (Ministry of Education), Department of Reproductive Endocrinology, Women’s Hospital, Zhejiang University School of Medicine, Hangzhou, China; 4 Shaoxing Maternity and Child Health Care Hospital, Shaoxing, China; 5 The International Peace Maternity and Child Health Hospital, School of Medicine, Shanghai Jiao Tong University, Shanghai, China; 6 Shanghai Key Laboratory of Embryo Original Diseases, Shanghai, China; 7 Departments of Obstetrics and Gynecology, Peking Union Medical College Hospital, Chinese Academy of Medical Sciences and Peking Union Medical College, Beijing, China; 8 Department of Pancreatic Surgery, Fudan University Shanghai Cancer Centre, Shanghai, China; 9 Key Laboratory of Disease Proteomics of Zhejiang Province, Department of Pathology and Pathophysiology, Zhejiang University School of Medicine, Hangzhou, China; 10 Department of Obstetrics and Gynecology, International Institutes of Medicine, the Fourth Affiliated Hospital of Zhejiang University School of Medicine, Yiwu, China

**Keywords:** Endocrine system and metabolic diseases, Metabolic disorders, Mechanisms of disease

## Abstract

Follicle-stimulating hormone (FSH) is involved in mammalian reproduction via binding to FSH receptor (FSHR). However, several studies have found that FSH and FSHR play important roles in extragonadal tissue. Here, we identified the expression of FSHR in human and mouse pancreatic islet β-cells. Blocking FSH signaling by Fshr knock-out led to impaired glucose tolerance owing to decreased insulin secretion, while high FSH levels caused insufficient insulin secretion as well. In vitro, we found that FSH orchestrated glucose-stimulated insulin secretion (GSIS) in a bell curve manner. Mechanistically, FSH primarily activates Gαs via FSHR, promoting the cAMP/protein kinase A (PKA) and calcium pathways to stimulate GSIS, whereas high FSH levels could activate Gαi to inhibit the cAMP/PKA pathway and the amplified effect on GSIS. Our results reveal the role of FSH in regulating pancreatic islet insulin secretion and provide avenues for future clinical investigation and therapeutic strategies for postmenopausal diabetes.

## Abstract (web summary)

Follicle-stimulating hormone (FSH) is involved in mammalian reproduction, but several studies have suggested a role of FSH and its receptor in extragonadal tissue. Here, the authors show that FSH orchestrates glucose-stimulated insulin secretion (GSIS) via its receptor on pancreatic β-cells, with pre-menopausal FSH levels dose-dependently promoting GSIS and postmenopausal FSH levels inhibiting this effect.

## Introduction

Follicle-stimulating hormone (FSH), a glycoprotein hormone secreted by basophils of the anterior pituitary lobe, primarily affects the gonads and regulates the reproductive process^1^. FSH acts on target cells via its receptor FSHR to transduce signals, triggering a cascade of biochemical signaling events^2^. Accumulating evidence has demonstrated that FSH and FSHR exert actions outside the reproductive tract, including in the adipose tissue^3,4^, hepatocytes^5–7^, osteoclasts^8,9^, and hippocampal and cortical neurons^10^, and play significant roles in postmenopausal female diseases. However, limited research has been conducted on the effect of different FSH level on the regulation of metabolism.

It is known that the pancreas is an important endocrine organ in regulating glucose homeostasis. Only one previous study found FSH and FSHR immunoreactivity in the pancreas of rat^11^. However, in their experiment, the method of whole pancreatic tissue incubation limited the reflection of actual insulin secretion capability, and, whether FSH affects the function of pancreatic islets remains unknown. In this study, we confirmed the expression of FSHR on human and mouse pancreatic islets for the first time, and, discovered a new extragonadal action of FSH on glucose-stimulated insulin secretion (GSIS). These results indicate the important role of FSH in maintaining glucose homeostasis, independent of the indirect action for FSH via estrogen^12^, providing a novel perspective of high FSH levels in the increased risk of developing diabetes mellitus in postmenopausal women.

## Results

### FSH regulates glucose-stimulated insulin secretion via FSHR

**Fig. 1:**
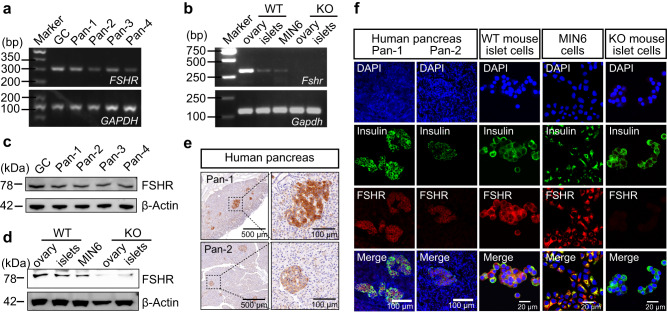
FSHR expression in human and mouse pancreatic β-cells and MIN6 cells. **a**, **b** The mRNA expression of FSHR in human female granulosa cells and pancreas (**a**), female mouse ovarian tissue and pancreatic islets, and MIN6 cells (**b**). *GAPDH* served as a loading control. **c**, **d** Protein expression of FSHR in human female granulosa cells and pancreas (**c**), female mouse ovarian tissues and pancreatic islets, and MIN6 cells (**d**). β-Actin served as a loading control. The ovary and pancreatic islets from Fshr^−/−^ (KO) mice as negative controls. GC, granulosa cells; Pan, pancreas. **e** Localization of FSHR in the human female pancreas. Scale bars, 500 µm (main images), 100 µm (magnified images). **f** Localization of FSHR (red) in the human female pancreas, mouse pancreatic islets single cells, and MIN6 cells. Insulin (green) was the marker of pancreatic β-cells. Nuclei (blue) were stained with DAPI. Scale bars, 100 µm (human pancreas), 20 µm (mouse islet cells, MIN6 cells, and NC). The experiments in (**a**–**f**) were performed twice independently. Source data are provided as a Source data file.

In our study, we first examined FSHR expression in human pancreas collected from patients undergoing pancreatic carcinoma surgery. FSHR was expressed in the human pancreas at both gene and protein levels in females and males (Fig. 1a, c, Supplementary Fig. 1a, b). FSHR was also identified in mouse pancreatic islets and mouse insulinoma cell line MIN6 (Fig. 1b, d). Further, immunohistochemical staining showed FSHR was localized in the cell membranes and cytoplasm of human pancreatic islets (Fig. 1e, Supplementary Fig. 1c). Immunofluorescence staining demonstrated colocalization of FSHR with insulin in human and mouse pancreatic islet β-cell and MIN6 cells (Fig. 1f, Supplementary Fig. 1d). Furthermore, FSHR was expressed in both the protein extracted from the cell membrane and cytoplasm of mouse pancreatic islets and MIN6 cells (Supplementary Fig. 2).

**Fig. 2:**
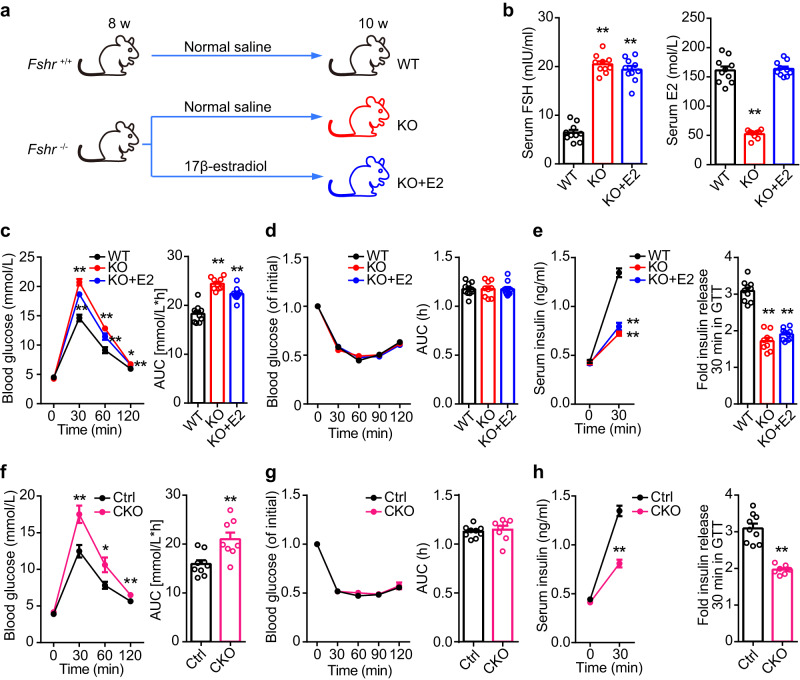
FSH, independent of estrogen, regulates GSIS via FSHR. **a** Schematic of Fshr^+/+^ (WT) and Fshr^−/−^ (KO) female mice subjected to different treatments. **b** Serum FSH and E2 levels in WT, KO and KO + E2 female mice with E2 supplement. n_WT_ = 10, n_KO_ = 9, n_KO+E2_ = 10. **P < 0.01 vs. WT, ^##^*P* < 0.01 vs. KO + E2. **c** Glucose tolerance test and AUC of female mice in different treatment groups. n_WT_ = 10, n_KO_ = 9, n_KO+E2_ = 10. *P < 0.05 vs. WT, **P < 0.01 vs. WT, ##*P* < 0.01 vs. KO + E2. **d** Insulin tolerance test and AUC of female mice in different treatment groups. n_WT_ = 10, n_KO_ = 9, n_KO+E2_ = 10. **e** Blood insulin levels at 0 min and 30 min after glucose injection and its fold changes of female mice with different treatments. n_WT_ = 10, n_KO_ = 9, n_KO+E2_ = 10. **P < 0.01 vs. WT. All the WT, KO and KO + E2 mice were performed the serum and metabolic tests at 10 weeks of age. **f** Glucose tolerance test and AUC of Ctrl (Fshr^f/f^) and CKO (Fshr^f/f^; Pdx1-Cre) female mice. n_Ctrl_ = 9, n_CKO_ = 8. *P < 0.05, **P < 0.01. **g** Insulin tolerance test and AUC of Ctrl and CKO female mice. n_Ctrl_ = 9, n_CKO_ = 8. **h** Blood insulin levels at 0 min and 30 min after glucose injection and its fold changes of Ctrl and CKO female mice. n_Ctrl_ = 9, n_CKO_ = 8. **P < 0.01. All the Ctrl and CKO mice were performed the serum and metabolic tests at 8 weeks of age. Data (**b**–**h**) were shown as mean ± s.e.m. and analyzed by one-way ANOVA (**b**–**e**) or unpaired two-tailed Student’s *t*-tests (**f**–**h**). Statistical details are in Supplementary Table 2. Source data are provided as a Source data file.

Insulin secretion is an important function of pancreatic islets. The expression of FSHR in pancreatic islets provides strong suggestive evidence that FSHR may be associated with the regulation of insulin secretion. To elucidate the role of FSH and its receptor in pancreatic islets, we established conventional Fshr^−/−^ (KO) mice. Fshr KO mice, including female and male mice, showed similar increases in body weight until 10 weeks of age (Supplementary Figs. 3a, 4a). Notably, Fshr KO female mice exhibited significantly impaired glucose tolerance (Fig. 2c). Conventional Fshr KO not only led to an increase in serum FSH levels but also caused a corresponding decrease in serum estrogen levels (Fig. 2b). To ensure that the glucose intolerance phenotype was exclusively affected by the FSHR signaling pathway, exogenous estrogen was administered to Fshr KO female mice to eliminate the adverse effects of endogenous low estrogen (Fig. 2a). Fshr KO females supplemented with estrogen also displayed impaired glucose tolerance (Fig. 2c). Further, we generated the mice with Fshr conditional knock-out (CKO) in pancreatic islet (Fshr^f/f^; Pdx1-Cre). The Fshr CKO females, which showed no significant alterations in serum FSH and estrogen levels (Supplementary Fig. 3g), exhibited glucose intolerance (Fig. 2f). Similarly, both male Fshr KO and CKO mice showed impaired glucose tolerance (Supplementary Fig. 4b, f).

**Fig. 3:**
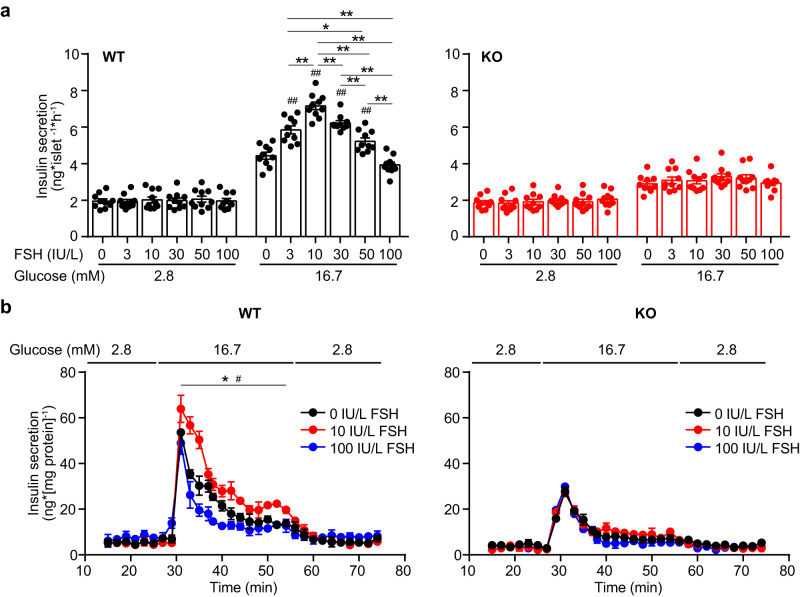
FSH regulates GSIS via FSHR in pancreatic islets. **a** GSIS in pancreatic islets from WT and KO female mice, with or without FSH, *n* = 10 repeats for each group. **P < 0.01; ^#^*P* < 0.05, ^##^*P* < 0.01 compared to 0 IU/L FSH group. **b** Perfusion analyses of dynamic GSIS in islets from WT, and KO female mice. Each islet sample was pooled from at least three animals, *n* = 3 technical replicates, *P < 0.05 0 IU/L vs. 10 IU/L, ^#^*P* < 0.05 10 IU/L vs. 100 IU/L. All the data were presented as mean ± s.e.m. and analyzed by one-way ANOVA. Statistical details are in Supplementary Table 2. Source data are provided as a Source data file.

Defective insulin action and secretion are two major causes of impaired glucose tolerance. Fshr KO mice with or without estrogen supplementation and Fshr CKO mice showed normal insulin sensitivity (Fig. 2d, g). The decreased serum insulin levels in response to glucose accounted for the impaired glucose tolerance (Fig. 2e, h). Correspondingly, in vitro static glucose-stimulated insulin secretion test using pancreatic islets isolated from Fshr KO mice demonstrated an insulin secretory defect in response to glucose (Fig. 3a, Supplementary Fig. 3b). Furthermore, perfusion analyses of dynamic GSIS in Fshr KO pancreatic islets were consistent with the results of static experiments (Fig. 3b). The number and morphology of pancreatic islets, and the ultrastructure of β-cells did not differ among the groups (Supplementary Fig. 3c–e). Taken together, these results suggest that blocking FSH signaling by ablating the Fshr gene could lead to impaired glucose tolerance due to decreased insulin secretion rather than changes in insulin action, indicating the necessity of FSH and FSHR in the maintenance of glucose homeostasis.

To investigate the effect of FSH on insulin secretion, we conducted an in vitro experiment to treat mouse pancreatic islets and MIN6 cell with different concentrations of FSH. Indeed, no significant differences were observed in Ins1 and Ins2 mRNA, and insulin content (Supplementary Fig. 5a, b, d, e) was observed in each group, implying that FSH did not affect insulin synthesis. FSH treatment alone, in the absence of glucose, had no independent stimulatory effect on insulin secretion in mouse pancreatic islets (Supplementary Fig. 5c, f). However, it is interesting that FSH orchestrates glucose-stimulated insulin secretion (GSIS) in a bell curve manner. In vitro, when the concentration of FSH was in the range of less than 10 IU/L, FSH promoted glucose-stimulated insulin secretion with increased FSH levels (Fig. 3a). However, when the concentration of FSH was higher than 10 IU/L, the amplified effect on GSIS was inhibited with increasing FSH concentration (Fig. 3). The regulatory effect of FSH on GSIS was abolished in FSHR knockout mouse islets in vitro (Fig. 3), indicating that the effects of FSH on GSIS depend on FSHR. A similar phenomenon was observed in males (Supplementary Fig. 6). In addition, when overexpression of FSHR in WT pancreatic islets and MIN6 cells, there was not a significant improvement of GSIS (Supplementary Fig. 7). These results suggested that FSHR is relative sufficient, and FSH level is important for GSIS regulation.

### High FSH level leads to impaired glucose tolerance

**Fig. 4:**
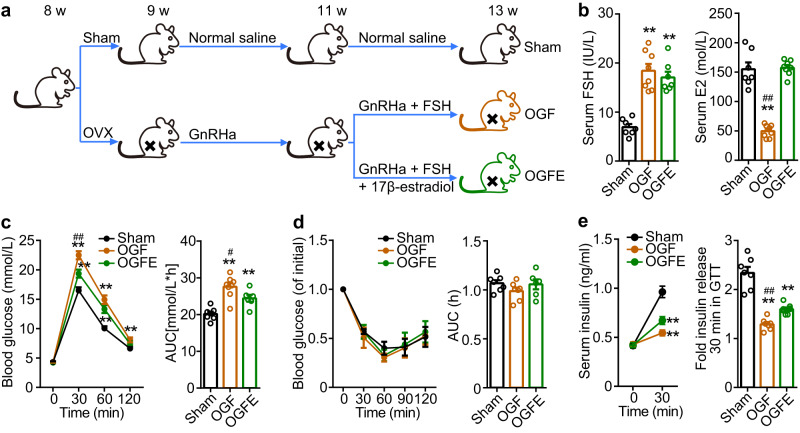
High circulating levels of FSH impaired glucose tolerance and insulin secretion. **a** Schematic of C57BL/6 female mice receiving different treatments. **b** Serum FSH and serum E2 levels in female mice with different treatments. n_Sham_ = 7, n_OGF_ = 8, n_OGFE_ = 7. **P < 0.01 vs. Sham, ^##^*P* < 0.01 vs. OGFE. **c** Glucose tolerance test and AUC of female mice in each group. n_Sham_ = 7, n_OGF_ = 8, n_OGFE_ = 6. *P < 0.05 vs. Sham, **P < 0.01 vs. Sham, ^#^*P* < 0.05 vs. OGFE. **d** Insulin tolerance test and AUC in female mice with different treatments. n_Sham_ = 7, n_OGF_ = 7, n_OGFE_ = 6. **e** Blood insulin levels at 0 min and 30 min after glucose injection and its fold changes of female mice with different treatments. *n* = 7 per group, **P < 0.01 vs. Sham, ^#^*P* < 0.05 vs. OGFE. All the female mice were performed the serum and metabolic tests at 13 weeks of age. Data (**b**–**e**) were shown as mean ± s.e.m. and analyzed by one-way ANOVA. Statistical details are in Supplementary Table 2. Source data are provided as a Source data file.

High serum FSH levels are a sign of menopause in females^13,14^. Our results suggest a strong link between elevated serum FSH levels and an increased risk of diabetes in postmenopausal women. This is consistent with clinical phenomenon in which epidemiological surveys have shown an increased risk of developing diabetes mellitus and metabolic syndrome in postmenopausal women^15–18^. We generated an ovariectomized (OVX) mouse model that mimicked the hormonal status of postmenopausal women. In OVX mice, both FSH and luteinizing hormone (LH) increased, whereas estrogen levels were decreased. OVX mice were injected with a gonadotropin-releasing hormone agonist (GnRHa) to induce pituitary desensitization, which inhibits both FSH and LH secretion, excluding the interference from high levels of LH. OVX + GnRHa mice were injected with exogenous FSH to mimic postmenopausal high serum FSH levels (OGF group). Similarly, to investigate the effects of FSH alone in vivo, OVX + GnRHa + FSH mice were administered an additional estrogen supplement to maintain relatively normal serum estrogen levels (OGFE group) (Fig. 4a, b). Meanwhile, there were no significant change of the expression of estrogen receptors (ERs) between sham and OGF groups (Supplementary Fig. 8). In addition, LH was also inhibited in OVX + GnRHa mice. We treated isolated pancreatic islets with LH, finding that LH had no significant effect on GSIS (Supplementary Fig. 9).

To examine the glucose metabolic state of the OVX mice subjected to different treatments, glucose and insulin tolerance tests were performed among these groups. Compared with the sham group (Sham), the OGF and OGFE groups, in which OVX mice have high levels of FSH, whether with estrogen supplements or not, showed overt glucose intolerance but normal insulin sensitivity (Fig. 4c, d). It is worth noting that the impaired glucose tolerance was more significant in the OGF group than in the OGFE group (Fig. 4c). OVX mice without estrogen supplementation exhibited a more severe decrease in insulin secretion (Fig. 4e). The number of pancreatic islet number (Supplementary Fig. 10b), and morphology (Supplementary Fig. 10c), and the ultrastructure of the β-cells (Supplementary Fig. 10d) were not significantly different among OVX groups. These results indicate that high levels of FSH alone lead to impaired glucose tolerance owing to insufficient insulin secretion. Previous studies have shown that a decline in estrogen levels explains the cause of postmenopausal diabetes to a certain extent^19–22^. In clinical practice, estrogen replacement therapy does not completely avoid this risk^23–26^. After hormone replacement therapy (HRT), the level of estrogen may improve; however, it is still difficult to recover the level of FSH to the pre-menopausal range^27^, which probably explains why estrogen replacement does not fully protect postmenopausal women from developing abnormal glucose tolerance. Previous studies showed that FSH/FSHR mediated the increased fat mass and fat redistribution in postmenopausal women, which may contribute to induce impaired glucose tolerance, indirectly, by decreased insulin sensitivity^3,4^. When fed with high-fat diet (HFD), the FSHR KO mice showed aggravated glucose intolerance (Supplementary Fig 11). However, we found that there was no significant difference of FSH level and FSHR expression in WT mice fed with HFD (Supplementary Fig. 12). This result indicated that, excluding the effects on insulin targeted organs, FSH/FSHR can directly regulate GSIS of pancreatic islets. Our findings revealed that FSH is a key but neglected factor in the regulation of glucose metabolism.

### FSH regulates GSIS via the cAMP/PKA and calcium pathway

**Fig. 5:**
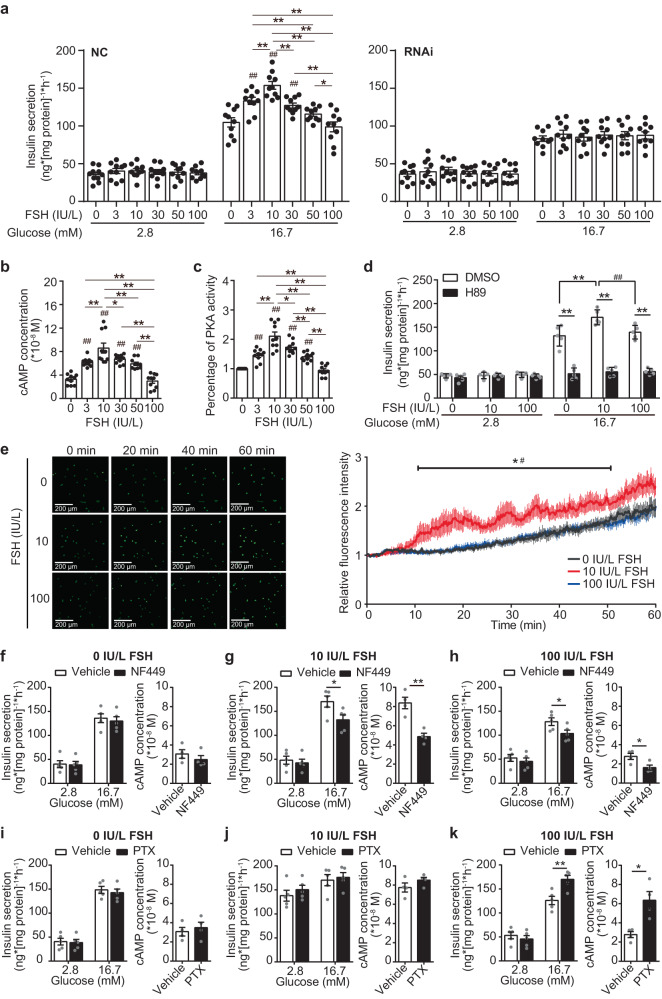
FSH orchestrates GSIS via the cAMP/PKA and Ca^2+^ pathway, which is controlled by Gαs and Gαi switch. **a** MIN6 cells were transfected with either the scramble (left) or the Fshr siRNA (right), followed by GSIS assays with different concentrations of FSH. *n* = 10 repeats each group, *P < 0.05, **P < 0.01; ^#^*P* < 0.05, ^##^*P* < 0.01 compared to 0 IU/L FSH group. **b**, **c** Intracellular cAMP concentration (**b**) and PKA activity (**c**) was measured in MIN6 cells after stimulated with 16.7 mM glucose and different concentrations of FSH for 1 h. *n* = 10 repeats each group, *P < 0.05, **P < 0.01; ^#^*P* < 0.05, ^##^*P* < 0.01 compared to 0 IU/L FSH group. **d** GSIS was tested in MIN6 cells treated with or without PKA inhibitor (H89, 10 μM). *n* = 5 repeats each group, *P < 0.05, **P < 0.01, ^#^*P* < 0.05. **e** Intracellular Ca^2+^ levels were measured by fluorescence of the Ca^2+^ dye Fluo-4 AM in MIN6 cells treated with or without FSH, addition of 16.7 mM glucose. *n* = 10 repeats each group, *P < 0.05 vs. 0 IU/L FSH, ^#^*P* < 0.05 vs. 100 IU/L FSH. **f**–**h** MIN6 cells were pre-treated with or without 10 μM NF449 (Gαs protein inhibitor) for 1 h, followed by GSIS assays and intracellular cAMP concentration measurements, under different concentrations of FSH. *n* = 5 repeats each group of GSIS assays, *n* = 4 repeats each group of intracellular cAMP concentration measurements, *P < 0.05, **P < 0.01. **i**–**k** GSIS assays and intracellular cAMP concentration measurements under different concentrations of FSH in MIN6 cells pre-treated with or without 100 ng/ml PTX (Gαi protein inhibitor), *n* = 5 repeats each group of GSIS assays, *n* = 4 repeats each group of intracellular cAMP concentration measurements, *P < 0.05, **P < 0.01. **a**–**k** Data were shown as mean ± s.e.m., *n* = 4 or 5 per group, analyzed by one-way ANOVA (**a**–**e**) and unpaired two-tailed Student’s *t*-tests (**f**–**k**). Statistical details are in Supplementary Table 2. Source data are provided as a Source data file.

To explore how FSH regulates intracellular insulin secretion, we established an MIN6 cell model, in which FSH had a similar concentration-dependent bell curve effect on GSIS (Fig. 5a). After FSHR knock down in MIN6 cells, we observed that the regulatory effect of FSH on GSIS was abolished (Fig. 5a), again demonstrating that the effects of FSH on GSIS depend on its receptor. Treated with increasing concentrations of FSH, the cell viability of MIN6 cells was not affected (Supplementary Fig. 13), excluding the effects of cellular activity on insulin secretion.

During GSIS, when transported into pancreatic islet β-cells, glucose undergoes a series of biochemical reactions and cooperation with ion channels, finally triggering the fusion of intracellular insulin-containing granules with the plasma membrane and thereby insulin secretion^28^. Exocytosis of insulin granules is a crucial step in insulin secretion^29^, an increase in intracellular Ca^2+^ concentration is the principal signal in this process^30,31^. In addition, in the glycolytic pathway, intracellular cAMP is produced from ATP by the action of adenylyl cyclase, which not only enhances in insulin granules exocytosis by activating cAMP-dependent protein kinase A (PKA)^32–34^, but also increases the sensitivity of β-cell exocytosis to calcium^35,36^. In our study, treatment with concentrations of FSH (<10 IU/L) markedly increased the intracellular cAMP levels in a concentration-dependent manner. In contrast, high concentrations of FSH (10–100 IU/L) decreased the intracellular cAMP levels in a concentration-dependent way (Fig. 5b). Intracellular PKA activity showed the same bell curve trend when treated with FSH (Fig. 5c). To further verify the role of PKA in FSH-regulated GSIS, we treated MIN6 cells with H89, PKI, and Rp-cAMPs, which are specific pharmacological PKA inhibitors, and found that FSH-regulated GSIS blocked when inhibiting PKA activity (Fig. 5d, Supplementary Fig. 14b). We further tested the downstream of the PKA pathway and found phosphorylated Kir6.2 and CaV1.2 increased after 10 IU/L FSH treatment and decreased at 100 IU/L (Supplementary Fig. 14e, f). Furthermore, calcium flux was measured using the Ca^2+^ dye Fluo-4 AM and real-time confocal imaging, which indicated that the Ca^2+^ level in 10 IU/L FSH-treated cells was higher than that in either 0 IU/L FSH-treated cells or 100 IU/L FSH-treated cells in high glucose buffer (Fig. 5e). In addition, MIN6 treated with verapamil (Ca^2+^ channel blocker) showed that the GSIS mediated by FSH was attenuated (Supplementary Fig. 14g), indicating that the origin of Ca^2+^ that increased by FSH was extracellular and flowed into cell via membrane binding Ca^2+^ channel. Taken together, these findings suggest that FSH regulates GSIS via the cAMP/PKA and calcium pathways.

### FSHR coupled Gαs/Gαi proteins are involved in FSH-regulated GSIS

FSHR is a specific G protein-coupled receptor (GPCR). Previous studies have shown that FSHR can regulate intracellular cAMP levels through coupled Gαs protein in ovarian granulosa cells or Gαi protein in Sertoli cells, respectively^37,38^. Therefore, after pretreatment of MIN6 cells with Gαs protein inhibitor NF449 or Gαi protein inhibitor PTX, we measured the effects of FSH on GSIS and intracellular cAMP content.

Pretreatment with Gαs inhibitor NF449 or Gαi inhibitor PTX did not affect the level of insulin secretion and the content of intracellular cAMP of MIN6 cells without FSH (Fig. 5f, i). In the 10 IU/L FSH and 100 IU/L FSH exposed groups, pretreatment with NF449 resulted in a significant decrease in the level of insulin secretion and the content of intracellular cAMP under 16.7 mM glucose (Fig. 5g, h). Pretreatment with PTX only increased the insulin secretion and the intracellular cAMP level under 16.7 mM glucose in the 100 IU/L FSH group (Fig. 5k), but no significant changes were observed in the 10 IU/L FSH group (Fig. 5j). These results demonstrated that in pancreatic β-cells, Gαs protein is involved in FSH-regulated GSIS, independent of the FSH concentration, while Gαi protein may participate in the regulation events only in a high concentration of FSH. Originally, each GPCR was thought to signal through a single cognate G protein class to initiate “canonical” signaling of the receptor. However, some studies have shown that receptors can also couple to more than one Gα protein^39–43^, and that, the agonist potency is greater for canonical versus non-canonical GPCR signaling^40^. In our study, in pancreatic β-cells, FSHR could couple with Gαs and Gαi proteins in an FSH dose-dependent manner, meaning that low concentrations of FSH primarily activate Gαs via FSHR, but high concentrations of FSH can also activate Gαi via FSHR. There was no significant change in FSHR expression in the pancreatic islets of the OVX model and MIN6 cells treated with different FSH concentrations (Supplementary Fig. 15), indicating that high FSH did not affect FSHR expression.

## Discussion

FSH primarily acts on the gonads and regulates the reproductive processes. Although the conventional idea has been expanded by the discovery of functional FSHR in bone cells, adipocytes, hepatocytes, and neurons, previous studies only have focused on postmenopausal high FSH level related to bone loss, visceral adiposity, or cognitive impairment^3,9,10^. In this study, we confirmed the expression of FSHR in human and mouse pancreatic islets for the first time, and revealed a novel and critical extragonadal action of FSH in the regulation of GSIS in pancreatic islets.

According the dissociation constant (Kd) of FSH, the FSH levels used in our study was within the range of its effective concentrations. The data indicated that pre-menopausal FSH levels could promote GSIS in a dose-dependent manner, however, the postmenopausal level of FSH inhibit the amplified effect on GSIS, showing a bell curve effect. In the clinic, patients with hypopituitarism always have a high prevalence of impaired glucose tolerance^44,45^, which could not be fully restored by growth hormone, cortisol, or thyroxine therapy^46,47^, suggesting that maybe other hormones, such as gonadotropin, should be considered. Our results demonstrate that a certain range of FSH is important for regulating insulin secretion, providing evidence to emphasize the role of FSH in the treatment of hypogonadotropic glucose intolerance.

We found that the high FSH levels which usually occurred in postmenopausal women induced glucose intolerance, that was previously mainly attributed to decreasing estrogen levels. Serum FSH levels undergo dramatic changes with age due to the loss of negative feedback from inhibin, as well as androgens and estrogens, in aging males and females, respectively^48–50^. In females, high serum FSH levels are one of the diagnostic signs of menopause^51^. Our previous clinical investigation of 9853 females, also showed that serum FSH levels in postmenopausal females were approximately 10-fold higher than those in their pre-menopausal counterparts^3^. In this study, we used the OVX + GnRHa + FSH mouse model to mimic the high FSH conditions of the postmenopausal period, excluding interference from high LH levels. In addition, the mice in another group were supplemented with estrogen to mimic clinical HRT and exclude the low estrogen effect. In clinic, some menopausal women still develop abnormal glucose metabolism despite being treated with HRT. Our results suggest that high FSH levels alone, independent of estrogen and ERs, could impair glucose tolerance by decreasing GSIS, which might be the potential explanation.

Inevitably, there were limitations to our study. The mice used in the OVX model were 8 weeks old; although sexually mature, older mice could better mimic the postmenopausal stage. In addition, pancreatic tissue from healthy individuals is difficult to obtain, we collected para-carcinoma tissues (non-tumorous normal tissues) from patients undergoing pancreatic carcinoma surgery to detect FSHR expression. Glycemic clamping in patients with hypopituitarism may provide more direct evidence for the role of FSH in improving GSIS. However, the incidence of hypopituitarism is rare. Recruiting adequate patients for the glycemic clamping could be taken consideration for further human study.

In summary, our study characterizes the novel role of FSH as a potential regulator of GSIS. For the first time, based on Fshr knock-out mouse models and in vitro experiments, we showed that the blockade of FSH signaling or a high FSH level can cause abnormal glucose tolerance due to insufficient insulin secretion. Our findings expand the understanding of the function of FSH in the regulation of metabolism and provide new avenues for future clinical investigation and therapeutic strategies for postmenopausal diabetes.

## Methods

### Human pancreas tissue

The human samples were obtained from the para-carcinoma tissue (non-tumorous normal tissues) of patients undergoing the pancreatic carcinoma surgery, the samples were paraffin-embedded for the immunohistochemistry and immunofluorescence analysis. All procedures involving human participants were reviewed and approved by the Ethic Committee of Fudan University Shanghai Cancer Center. Written informed consents were obtained from all participants. Participants didn’t receive cash remuneration. The study conformed to the ethical guidelines of the 1975 Declaration of Helsinki.

### Human ovarian granulosa cells

Ovarian granulosa cells (GCs) were obtained from follicular fluid of patients undergoing in vitro fertilization (IVF) procedures. The GCs, suspended in follicular fluid, were washed twice by centrifugation at 200 × *g* for 10 min at room temperature, and cultured in DMEM medium for the further experiment. All procedures involving human participants were reviewed and approved by the medical scientific research ethic committee of International Peace Maternity and Child Health Hospital. Written informed consents were obtained from all participants. Participants didn’t receive cash remuneration. The study conformed to the ethical guidelines of the 1975 Declaration of Helsinki.

### Animal care

All animals in our research were received humane care and maintained at Zhejiang University Animal Center under a 12/12 h light-dark cycle in a climate-controlled clean room with humidity range of 40–70% and temperature range of 25 ± 0.5 °C. Animals were allowed *ad libitum* access to a standard diet (10 kcal% fat, 20 kcal% protein, and 70 kcal% carbohydrate; Research diet, D10001) and water. At endpoint of animal experiments, mice were euthanized by CO_2_ asphyxiation. All animal protocols were approved by the Laboratory Animal Welfare and Ethics Committee of Zhejiang University and were consistent with the ARRIVE guidelines.

### OVX mice

Eight-week-old female C57BL/6 J mice were obtained from Shanghai SLAC Laboratory Animal Company. Female mice were OVX under sodium pentobarbital (Sigma) anesthesia (50 mg/kg body weight) and divided into three groups, (1) Sham: sham-operated; (2) OGF: OVX mice were initially injected with GnRHa (0.5 μg/day, Triptorelin Acetate Injection, Ferring) for 2 weeks, then injected with human recombinant FSH (0.15 IU/day, Gonal-F, Serono) and GnRHa (0.5 μg/day) for another 2 weeks; (3) OGFE: OVX mice were initially injected with GnRHa (0.5 μg/day) for 2 weeks, and injected with human recombinant FSH (0.15 IU/day), 17β-estradiol (0.6 μg/day, Sigma), and GnRHa (0.5 μg/day) for another 2 weeks. The surgical and drug treatment were adapted from protocols described previously^3,52^.

### Fshr konockout (KO) mice

Fshr^+/−^ mice were purchased from Beijing Biocytogen Company and intercrossed to produce Fshr^+/+^ (WT) and Fshr^−/−^ (KO) mice. The female mice were divided into three groups, (1) WT: Fshr^+/+^ mice were injected with saline daily for 2 weeks; (2) KO: Fshr^−/−^ mice were injected with saline daily for 2 weeks; (3) KO + E2: Fshr^−/−^ mice were injected with 17β-estradiol (0.6 μg, Sigma) daily for 2 weeks.

### Conditional Fshr KO mice

To achieve the pancreatic islet conditional inactivation of Fshr, we created mice with conditional Fshr KO by crossbreeding Fshr (flox/flox) mice (purchased from GemPharmatech Co.) with Pdx1-Cre mice (purchased from GemPharmatech Co.). Floxed mice without Pdx1-Cre served as the wild-type (WT) control.

### Cell culture

The MIN6 cell line used in our study was a cancer cell line and established from mice insulinomas. MIN6 cells obtained from Zhuo-Xian Meng’s lab at the Zhejiang University School of Medicine. MIN6 cells exhibits characteristics of glucose-stimulated insulin secretion similar to the normal pancreatic β-cells^53^. MIN6 cells (passages 15–30) were grown in a DMEM medium containing 15% FBS (vol./vol.), 25 mmol/l glucose, and 50 μmol/l 2-mercaptoethanol^54^ under a humidified atmosphere of 95% air and 5% CO_2_ at 37 °C.

### In vivo metabolic test

For the glucose tolerance test (GTT), after overnight starvation (16 h), mice were administered glucose (2 g/kg body weight) intraperitoneally. Blood glucose levels were measured at 0, 30, 60, and 120 min post-injection using the glucometer (Roche Diagnostics Accu-Chek). Serum was collected at 0 and 30 min after the glucose injection from tails for the insulin level measurement by ELISA kit (Crystal Chem,). For the insulin tolerance test (ITT), mice were starved for 4 h and injected intraperitoneally with insulin (0.7 U/kg body weight). Glucose levels were measured at 0, 30, 60, 90, and 120 min after injection.

### Pancreatic islet isolation and culture

Mouse pancreatic islets were isolated by collagenase IV (2 mg/ml; Worthington) digestion, separated by density gradient centrifugation, and handpicked under a stereomicroscope^55^. Islets were cultured in a standard RPMI medium supplemented with 10% FBS. For islet single-cell isolation, intact islets were incubated in Accutase (Gibco) at room temperature for 20 min with gentle intermittent mixing by pipetting^56^. The cells were then plated on glass coverslips pre-coated with poly-L-lysine solution (Sigma) and used for culture and staining.

### Glucose-stimulated insulin secretion (GSIS)

For indicated experiments, following preincubation for 30 min in glucose-free Krebs-Ringer bicarbonate buffer (KRBB), ten islets (size-matched for each batch) or MIN6 cells were incubated for 1 h in KRBB containing 2.8 mmol/L or 16.7 mmol/L glucose, supplemented with or without FSH and LH^54^. The supernatant fractions were obtained for the measurement of insulin. The insulin content of cells or islets was extracted overnight in acid-ethanol solution (70% [vol./vol.] ethanol, 0.18 N hydrochloride acid) at 4 °C. The insulin levels were measured using a mouse insulin ELISA kit.

For perfusion islet experiment, at least 100 hand-picked pancreatic islets from each mouse were placed into the Bio-Gel P-4 bead chambers for insulin secretion analysis. An automated perfusion system (BioRep, Miami, USA) was used in the assay. The islets were equilibrated in Krebs-Ringer bicarbonate HEPES buffer (KRBH) with 2.8 mmol/L glucose for 30 min and then perfused as follows (flow rate 0.1 mL/min): KRBH with 2.8 mmol/L glucose for 25 min, KRBH with 16.8 mmol/L glucose (containing 0 IU/L, 10 IU/L, or 100 IU/L FSH, respectively) for 30 min, KRBH with 2.8 mmol/L glucose for 20 min. The perfusate was collected every minute and used for insulin measurement. The total protein content of each sample was also detected for standardizing insulin secretion^54^.

### Pancreatic islet morphology and electron microscopy

The pancreas was paraffin-embedded and sectioned at 4 μm intervals, followed by hematoxylin and eosin (H&E) staining. Isolated islets were fixed in 2.5% glutaraldehyde and postfixed in 1% osmium tetroxide and then stained with aqueous 2% uranyl acetate for 30 min, dehydrated, and embedded in epoxy resin. Sections were stained with uranyl acetate and lead citrate. Samples were examined under TECNAI 10 transmission electron microscope (Philips).

### Quantitative real-time PCR

Total RNA was isolated using the RNAiso Reagent (Takara) and cDNA was prepared using the PrimeScript RT kit (Takara). Quantitative real-time PCR (qPCR) was performed with the SYBR Premix Ex Taq (Tli RNaseH Plus) system (Takara). qPCR was carried out with specific primers on ABI Prism 7900HT (Applied Biosystems). All primer sequences are shown in Supplementary Table 1.

### Western blot analysis

The tissue or cell samples were homogenized in RIPA buffer containing protease inhibitor cocktail. Protein per sample were loaded and separated in a 10% SDS-PAGE gel. The separated samples were transferred to nitrocellulose membranes. After blocking with 5% nonfat milk, the membranes were incubated to the primary antibodies anti-FSHR (1:500; Abcam, ab75200), anti-PKA (1:1000; CST, #4782), anti-Kir6.2 (1:1000; Santa Cruz, sc390104), anti-pKir6.2 (1:1000; Invitrogen, PA5-40157), and anti-CaV1.2 (1:1000; Abcam, ab270987), anti-pCaV1.2 (1:1000; Invitrogen, PA5-64748), anti-ERα (1:1000; Invitrogen, MA1 − 80216), anti-ERβ (1:1000; Invitrogen, PA1 − 311), anti-GPCR30 (1:1000; Abcam, ab260033), anti-β-Actin (1:10,000; Proteintech, 66009-1-lg) or anti-GAPDH (1:10,000; Proteintech, 60004-1-lg) at 4 °C overnight. Following incubation with the corresponding secondary antibodies, images were taken by BIO-RAD ChemiDoc MP and analyzed by Image J 1.52a software.

### Immunohistochemistry and immunofluorescence staining

For immunohistochemistry, tissue samples were sectioned at 4 μm intervals, blocked in 1% BSA, and incubated with anti-FSHR (1:100; Abcam, ab75200) antibody at 4 °C overnight. Tissue sections were then washed with PBS and incubated with secondary antibody conjugated with HRP (1:200; Sangon Biotech, D110058). Immunostained sections were characterized quantitatively by digital image analysis using Image Pro-Plus 6.0 (Zeiss LSM 510).

For immunofluorescence, fixed and permeabilized tissue sections, mouse pancreatic islet single cells, and MIN6 cells were blocked with 1% BSA and then incubated with anti-FSHR (1:100; Abcam, ab75200), and anti-insulin (1:500; CST, #8138) antibodies at 4 °C overnight. After washing with PBS, the sections and cells were incubated with fluorescent-conjugated secondary antibodies (1:500; Invitrogen, A21241, A11034). Nuclei were stained with DAPI (Abcam, ab228549). All immunofluorescence images were acquired by laser scanning confocal microscope (Zeiss).

### RNA interference

Small interfering RNA (siRNA) duplexes were designed and chemically synthesized. The sense sequence for Fshr siRNA was 5’-CCTCTGAACTTCATCCAAT-3’, and the sense sequence for silencer negative siRNA was 5’-GGCUCUAGAAAAGCCUAUGC-3’. Using Lipofectamine RNAiMAX Transfection Reagent (Invitrogen), MIN6 cells were transfected with 5 nM Fshr siRNA or 5 nM silencer negative siRNA.

### Cell viability assay

MIN6 cells were seeded in a 96-well plate with 5000 cells/well, and treated with a fresh culture medium with different concentrations (0–100 IU/L) of FSH. Cell viability was assessed by a Cell Counting Kit-8 (CCK-8; Dojindo Molecular Technologies) according to the manufacturer’s instructions. Briefly, after treatment, the CCK-8 solution was added to the culture medium and incubated at 37 °C for 1 h. The absorbance was read at 450 nm with a microplate reader (Bio-Rad). Cell viability was calculated by (experimental group absorbance value/control group absorbance value) × 100%.

### cAMP assay

Following pre-incubated in KRBB without glucose for 30 min, MIN6 were incubated in KRBB supplemented with 16.7 mM glucose for 1 h in the absence or presence of FSH, with IBMX (100 μM), an inhibitor of phosphodiesterase. After incubation, cells were harvested and washed in PBS three times. All the samples were dissected and sonicated in cell lysis buffer on ice, and freeze/thaw cycle twice before centrifugation at 600 × *g* for 10 min. Supernatants were collected for cAMP assays. cAMP levels were measured using cAMP Assay kits (R&D Systems) according to the manufacturer’s protocols. All samples and standards were assayed in duplicate, and the results were averaged.

### PKA activity assay

The MIN6 cells were incubated in KRBB without glucose for 30 min, then incubated in KRBB with 16.7 mM glucose and different concentrations of FSH for 1 h. After treatment, the cells were harvested, washed in PBS, resuspended in PKA extraction buffer, and incubated on ice for homogenization. Samples were cleared by centrifugation and supernatants were used for the PKA assay. The qualitative activity of PKA was measured by PepTag non-radioactive protein kinase assays (Promega) according to the manufacturer’s description.

### Intracellular Ca^2+^ imaging

MIN6 cells (1 × 10^5^ cells per dish) were seeded and allowed to attach to cover slides in 35 mm culture dishes for 24 h. Cells were then pre-incubated for 30 min in KRBB containing 5 μmol/l Fluo-4 AM (Invitrogen), washed, and equilibrated for a further 15 min in KRB buffer without Fluo‐4 AM^57^. Then changed the medium to KRBB, which contains 16.7 mM glucose and with or without FSH, real‐time fluorescence was acquired every 10 s for 60 min by confocal microscope (excitation = 485 nm; emission = 520 nm) and analyzed by Image J software.

### Statistical analysis

All data are presented as mean ± s.e.m. Statistical significance was assessed by either unpaired two-tailed Student’s *t*-test (two-group comparison) or one-way ANOVA with LSD post hoc test (more than two groups), using SPSS 22.0 software. Differences with *P*  <  0.05 were considered to be significant.

### Reporting summary

Further information on research design is available in the Nature Portfolio Reporting Summary linked to this article.

### Supplementary information

Supplementary Information

Reporting Summary

### Source data

Source Data

## Data Availability

All datasets generated in this study are provided in the Supplementary Information/Source data file. Source data are provided with this paper.
