# Supplementary material for: Follicle-stimulating hormone orchestrates glucose-stimulated insulin secretion of pancreatic islets
Source: Nat Commun. 2023 Nov 1;14:6991. doi: 10.1038/s41467-023-42801-6 (PMC10620214; doi:10.1038/s41467-023-42801-6)

Supplementary Fig. 1

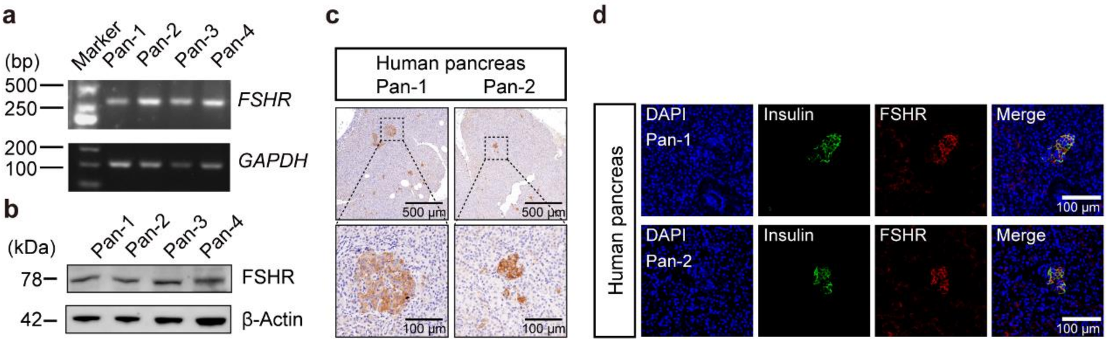

Supplementary Figure 1. Expression of FSHR in human male pancreatic β-cells. (**a**) mRNA expression of FSHR in the pancreas of the human male. *GAPDH* served as a loading control. (**b**) Protein expression of FSHR in pancreas of the human male. β-Actin served as a loading control. Pan, pancreas. (**c**) Localization of FSHR in the human male pancreas by immunohistochemistry. Scale bars, 500 μm (main images), 100 μm (magnified images). (**d**) Localization of FSHR (red) in the human male pancreas by immunofluorescence, Insulin (green) was the marker of pancreatic β-cell, and nuclei (blue) were stained with DAPI. Scale bars, 100 μm. The experiments in **a-d** were performed twice independently. Source data are provided as a Source Data file.

Supplementary Fig. 2

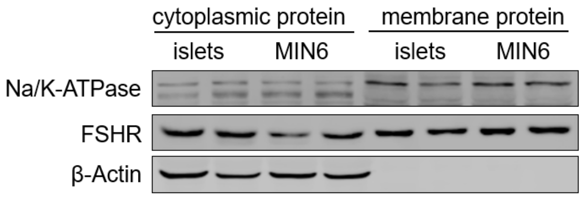

Supplementary Figure 2. FSHR expressed in both the cell membrane and the cytoplasm.

FSHR expression in cytoplasmic and membrane of cells from mouse pancreatic islets and MIN6 cells. The experiment was repeated 3 times with similar results.

Supplementary Fig. 3

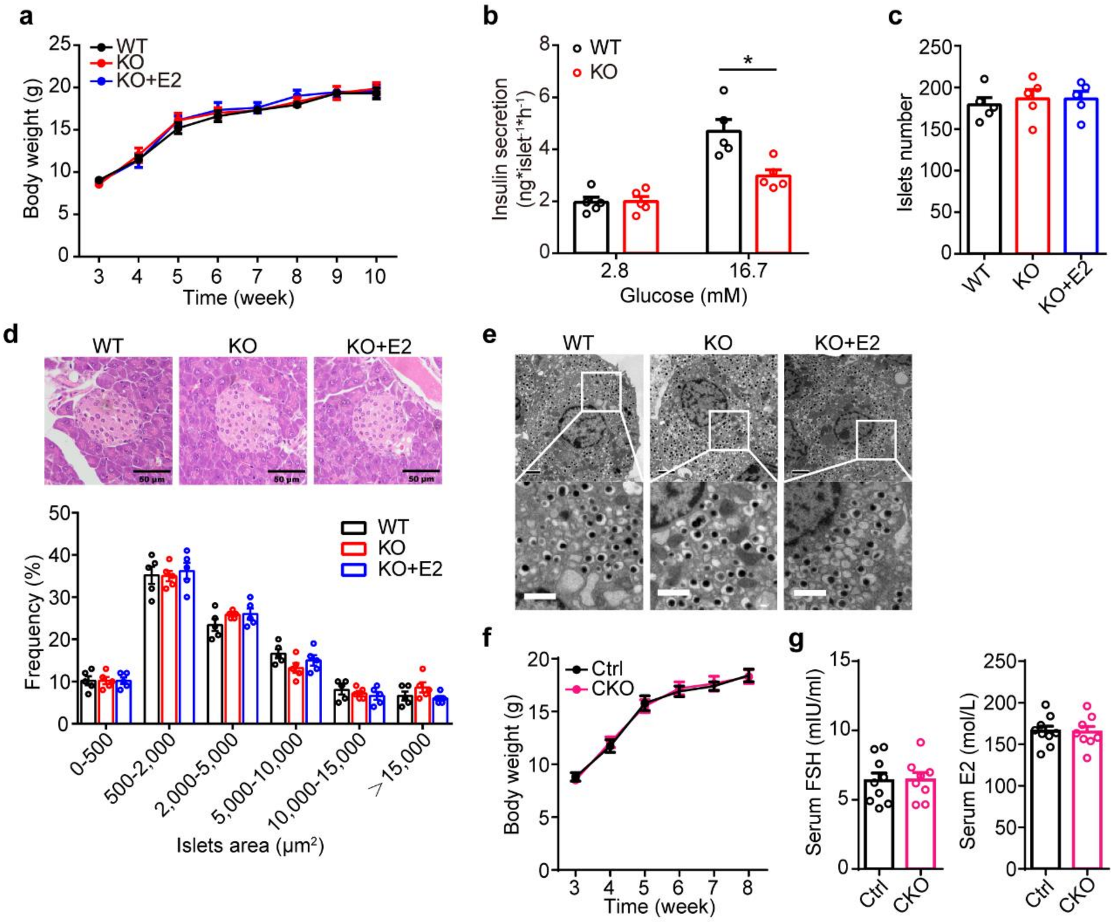

Supplementary Figure 3. The number and morphology of pancreatic islets in FSHR knockout mouse.

(**a**) Body weight of the WT, KO, and KO+E2 female mice. (**b**) GSIS in isolated islets from 10-week-old female WT and KO mice, n = 5 per group. (**c**) The number of pancreatic islets in each group, n = 5 per group. (**d**) Hematoxylin and eosin (H&E) staining in paraffin-embedded sections of the pancreas and the profiling of islet size distribution. Scare bars, 50 μm. Shown are representative images from 5 mice per group with similar results. (**e**) Electron microscopic images of pancreatic β-cells. Scare bars, 1 μm. (**f**) Body weight of the Ctrl, and CKO female mice. (**g**) Serum FSH and E_2 levels of the Ctrl, and CKO female mice. Data were shown as mean ± s.e.m., analyzed by unpaired two-tailed Student's *t*-tests **(b, f, g)** and one-way ANOVA (**a, c, d**), *$P < 0.05$. Statistical details are in Supplementary Table 2. Source data are provided as a Source Data file.

Supplementary Fig. 4

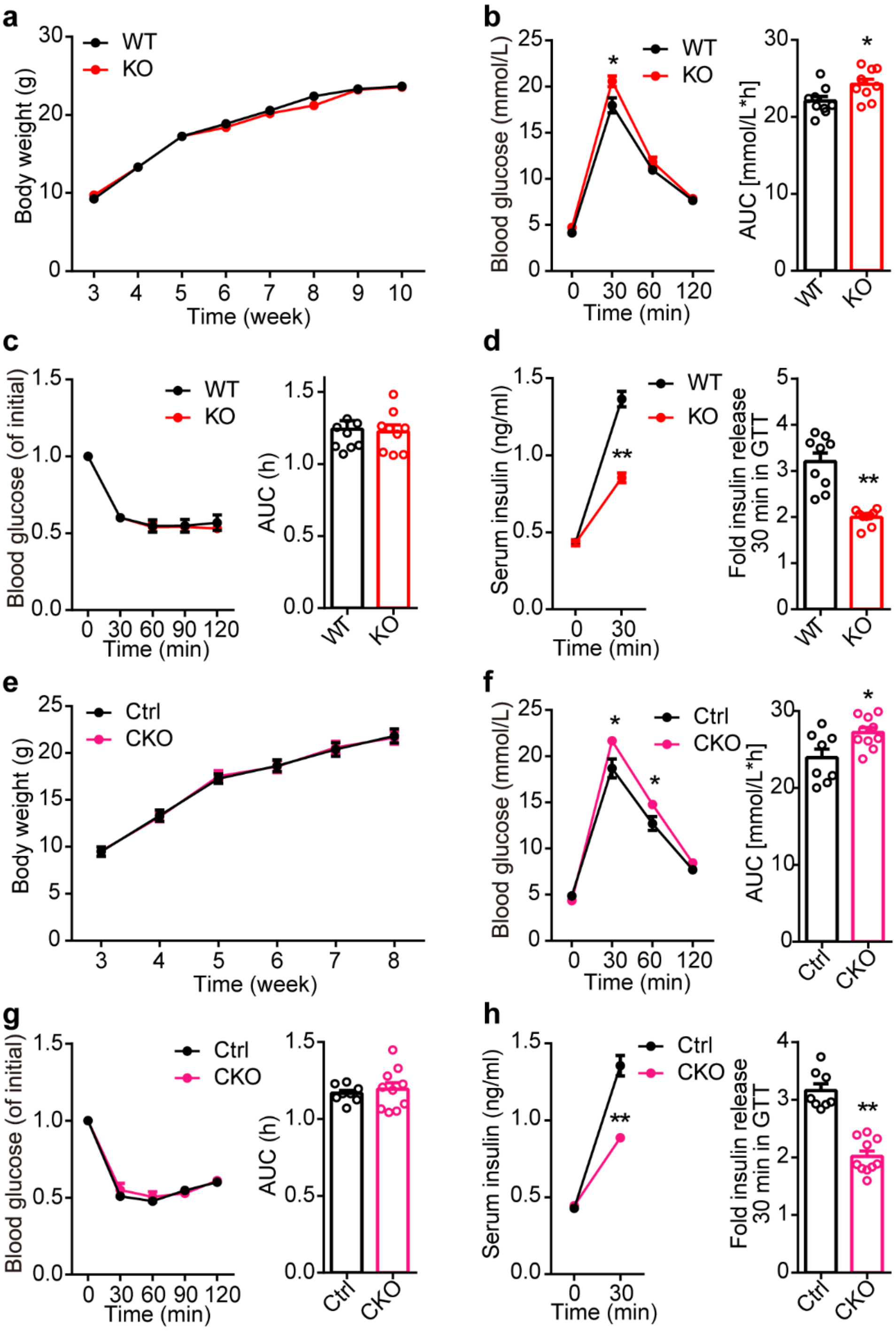

Supplementary Figure 4. FSHR knockout lead to impaired glucose tolerance and insulin secretion in male mice.

(**a**) Body weight of $Fshr^{+/+}$ (WT) and $Fshr^{-/-}$ (KO) male mice. (**b**) Glucose tolerance test and AUC of WT and KO male mice in different treatment groups. $n_{WT} = 9$, $n_{KO} = 9$. *$P < 0.05$. (**c**) Insulin tolerance test and AUC of WT and KO male mice in different treatment groups. $n_{WT} = 9$, $n_{KO} = 9$. (**d**) Blood insulin levels at 0 min and 30 min after

glucose injection and its fold changes of WT and KO male mice with different treatments. $n_{WT} = 9$, $n_{KO} = 9$. $^{**}P < 0.01$. All the WT and KO male mice were performed the serum and metabolic tests at 10 weeks of age. (**e**) Body weight of Ctrl ($Fshr^{f/f}$) and CKO ($Fshr^{f/f}$; Pdx1-Cre) male mice. (**f**) Glucose tolerance test and AUC of Ctrl and CKO male mice in different treatment groups. $n_{Ctrl} = 8$, $n_{CKO} = 10$. $^{*}P < 0.05$. (**g**) Insulin tolerance test and AUC of Ctrl and CKO male mice in different treatment groups. $n_{Ctrl} = 8$, $n_{CKO} = 10$. (**e**) Blood insulin levels at 0 min and 30 min after glucose injection and its fold changes of Ctrl and CKO male mice with different treatments. $n_{Ctrl} = 8$, $n_{CKO} = 10$. $^{**}P < 0.01$. All the Ctrl and CKO male mice were performed the serum and metabolic tests at 8 weeks of age. Data (**a-h**) were shown as mean ± s.e.m. and analyzed by unpaired two-tailed Student's *t*-tests. Statistical details are in Supplementary Table 2. Source data are provided as a Source Data file.

Supplementary Fig. 5

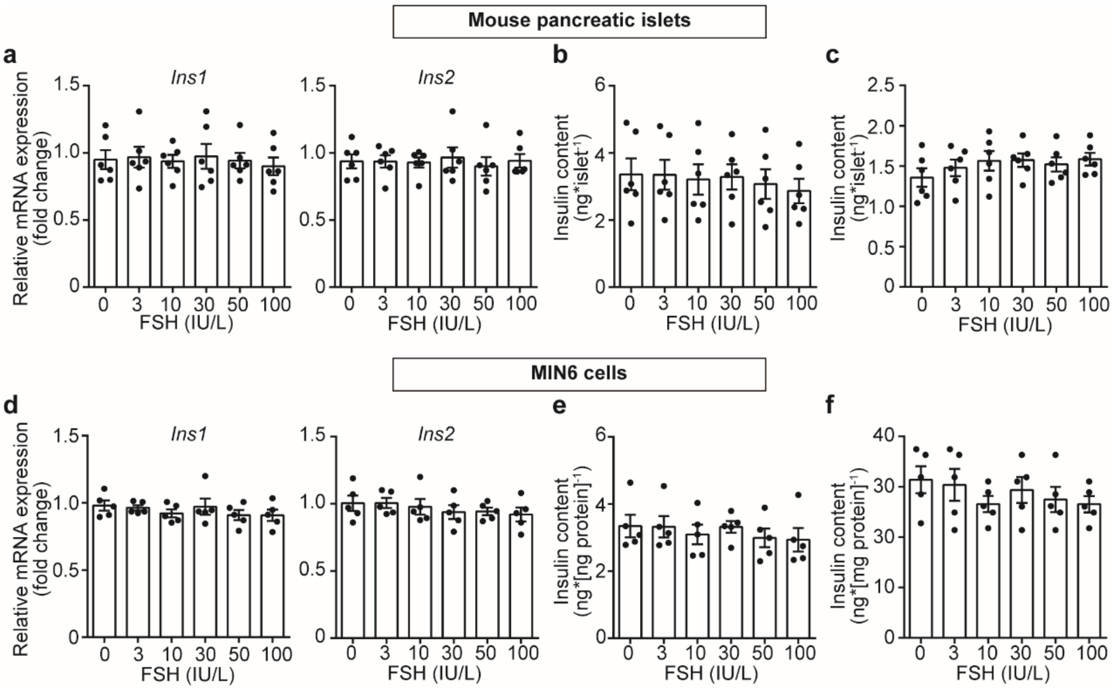

Supplementary Figure 5. FSH, alone, did not stimulate insulin synthesis and secretion.

(**a-b**) Ins1, Ins2 mRNA expression (**a**) and intracellular insulin content (**b**) in mouse pancreatic islets treated with or without FSH for 48 hours. (**c**) Supernatant insulin secretion level in mouse pancreatic islets treated with or without FSH for 1 hour, under 0 mM glucose. (**d-e**) Ins1, Ins2 mRNA expression (**d**) and intracellular insulin content (**e**) in MIN6 cells treated with or without FSH for 48 hours. (**f**) Supernatant insulin secretion level in MIN6 cells treated with or without FSH for 1 hour, under 0 mM glucose. Data were shown as mean ± s.e.m., n = 6 per group **(a-c)**, n = 5 per group **(d-f)**. Statistical details are in Supplementary Table 2. Source data are provided as a Source Data file.

Supplementary Fig. 6

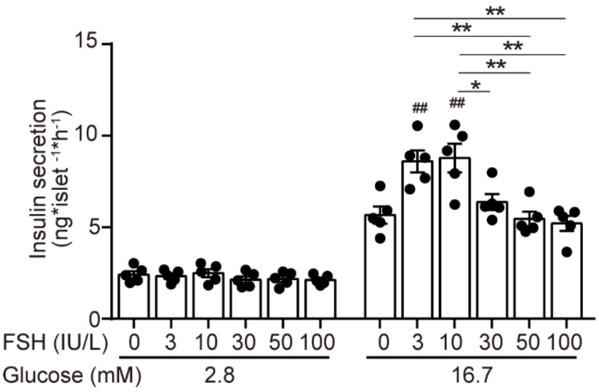

Supplementary Figure 6. FSH regulated GSIS in a bell curve manner in pancreatic islets of male mice.

GSIS in pancreatic islets from 10-week-old C57BL/6 male mice, with or without FSH, n = 5 for each group. $*P < 0.05$, $**P < 0.01$; $\#P < 0.05$ compared to 0 IU/L FSH group. Data were presented as mean ± s.e.m. and analyzed by one-way ANOVA. Statistical details are in Supplementary Table 2. Source data are provided as a Source Data file.

Supplementary Fig. 7

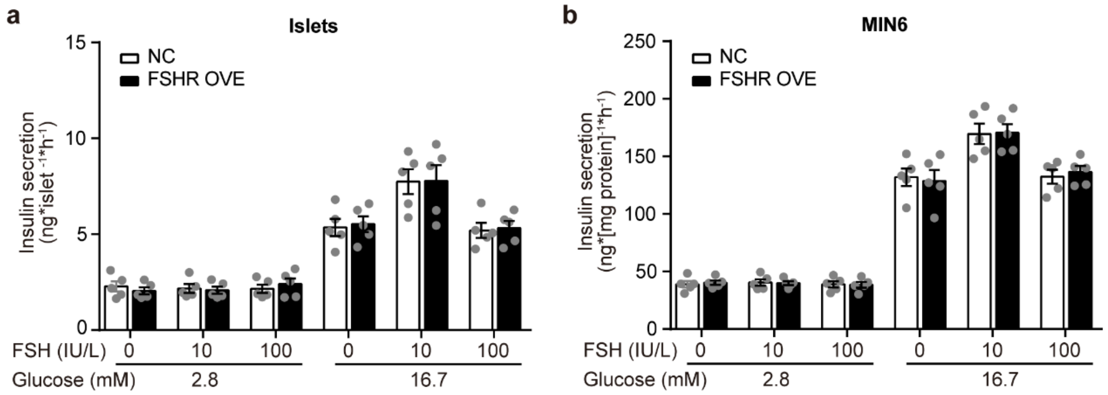

Supplementary Figure 7. Overexpression of FSHR in WT pancreatic islets and MIN6 cells, there was not a significant improvement of GSIS.

Pancreatic islets (a) and MIN6 cells (b) were transfected with either the scramble (NC) or the FSHR overexpression lentivirus (FSHR OVE), followed by GSIS assays with different concentrations of FSH. Data were shown as mean ± s.e.m., n = 5 per group. Statistical details are in Supplementary Table 2. Source data are provided as a Source Data file.

Supplementary Fig. 8

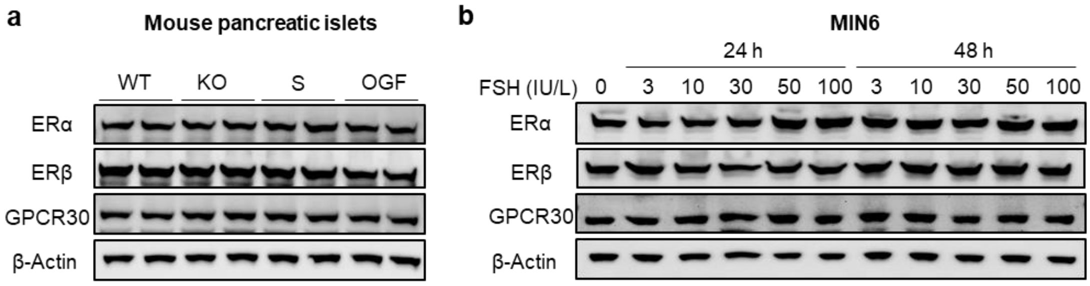

Supplementary Figure 8. The expression of ERs was not altered on the pancreatic islets from FSHR KO mice and FSH-treated MIN6 cells.

(a) The ERs expression on the pancreatic islets from WT and FSHR KO mice. **(b)** The ERs expression on the MIN6 cells with different concentrations of FSH treatment. The experiments were repeated 3 times with similar results.

Supplementary Fig. 9

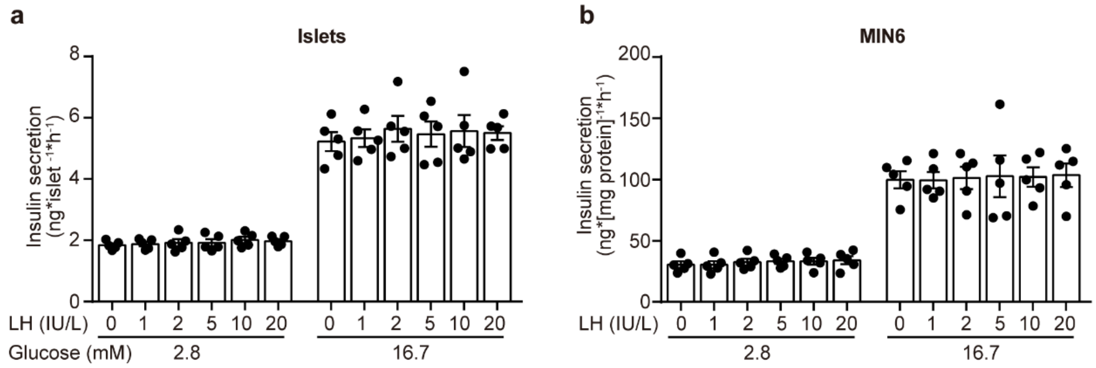

Supplementary Figure 9. LH did not affect glucose stimulated insulin secretion. (**a**) GSIS in pancreatic islets from 10-week-old C57BL/6 female mice, with or without LH, n = 5 for each group. (**b**) GSIS in MIN6 cells, with or without LH, n = 5 for each group. Data were presented as mean ± s.e.m. and analyzed by one-way ANOVA. Statistical details are in Supplementary Table 2. Source data are provided as a Source Data file.

Supplementary Fig. 10

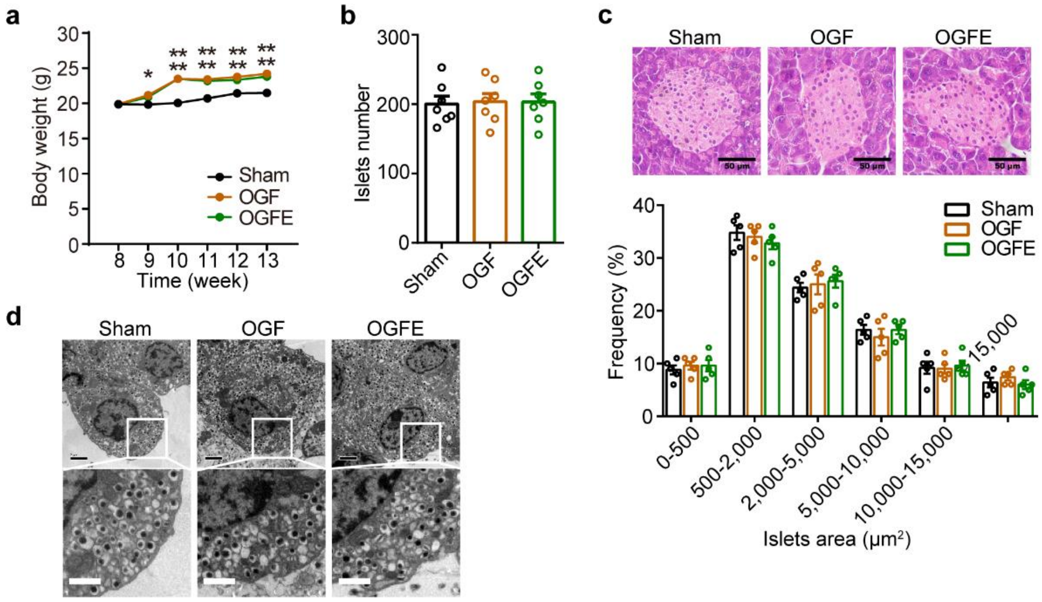

Supplementary Figure 10. Pancreatic islet number, morphology, and ultrastructure in the OVX mouse model.

(**a**) The body weight of mice in Sham, OGF and OGFE group. (**b**) The number of pancreatic islets of each group. Data are mean ± s.e.m., n = 7 per group. (**c**) H&E staining in paraffin-embedded sections of the pancreas and the profiling of islet size distribution, scare bars, 50 μm. Shown are representative images from 5 mice per group with similar results. (**d**) Electron microscopic images of pancreatic β-cells. scare bars, 1 μm. Statistical details are in Supplementary Table 2. Source data are provided as a Source Data file.

Supplementary Fig. 11

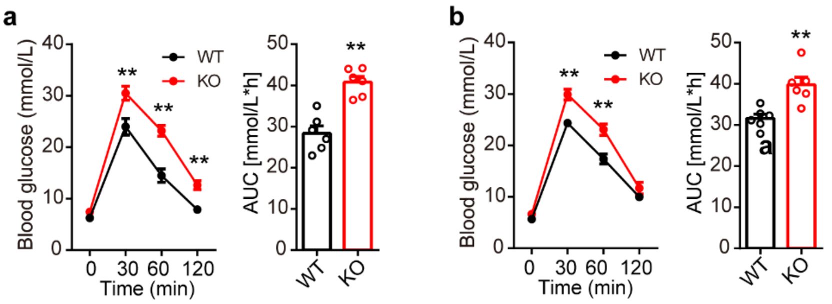

Supplementary Figure 11. HFD aggravated glucose intolerance in FSHR KO mice. **(a)** Glucose tolerance test and AUC of WT and KO female mice fed with HFD. $n_{WT} = 6$, $n_{KO} = 6$. **(b)** Glucose tolerance test and AUC of WT and KO male mice fed with HFD. $n_{WT} = 6$, $n_{KO} = 6$. **$P < 0.01$, data were shown as mean ± s.e.m. and analyzed by unpaired two-tailed Student's *t*-tests. Statistical details are in Supplementary Table 2. Source data are provided as a Source Data file.

Supplementary Figure 12

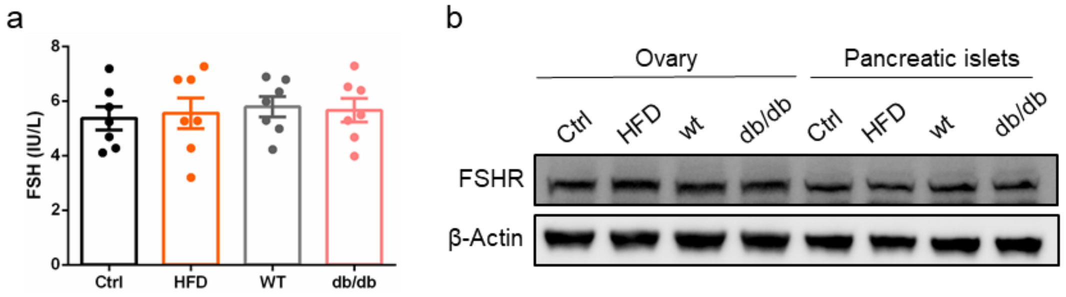

Supplementary Figure 12. The serum FSH level and FSHR expression and was not altered in HFD mice and db/db mice.

(a) Serum FSH levels in 20-week-old C57BL/6 female mice (Ctrl), HFD fed 20-week-old C57BL/6 female mice (HFD), 10-week-old WT and db/db female mice (db/db). Data were presented as mean ± s.e.m., n = 7 per group. **(b)** FSHR expression in ovary and pancreatic islets from Ctrl, HFD, WT, and db/db mice. The experiments were performed 3 times independently with similar results. Statistical details are in Supplementary Table 2. Source data are provided as a Source Data file.

Supplementary Fig. 13

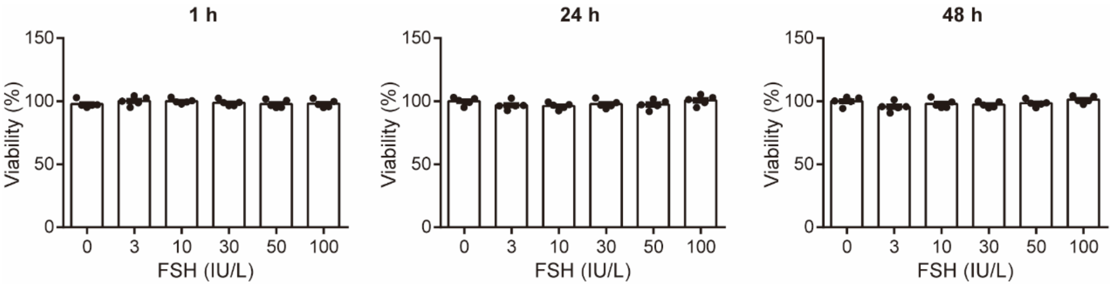

Supplementary Figure 13. The effect of FSH on cell viability in MIN6 cells. Cell viability validation of MIN6 cells treated with different concentrations of FSH for 1 hour, 24 hours, or 48 hours. Data were shown as mean ± s.e.m., n = 5 per group. Statistical details are in Supplementary Table 2. Source data are provided as a Source Data file.

Supplementary Fig. 14

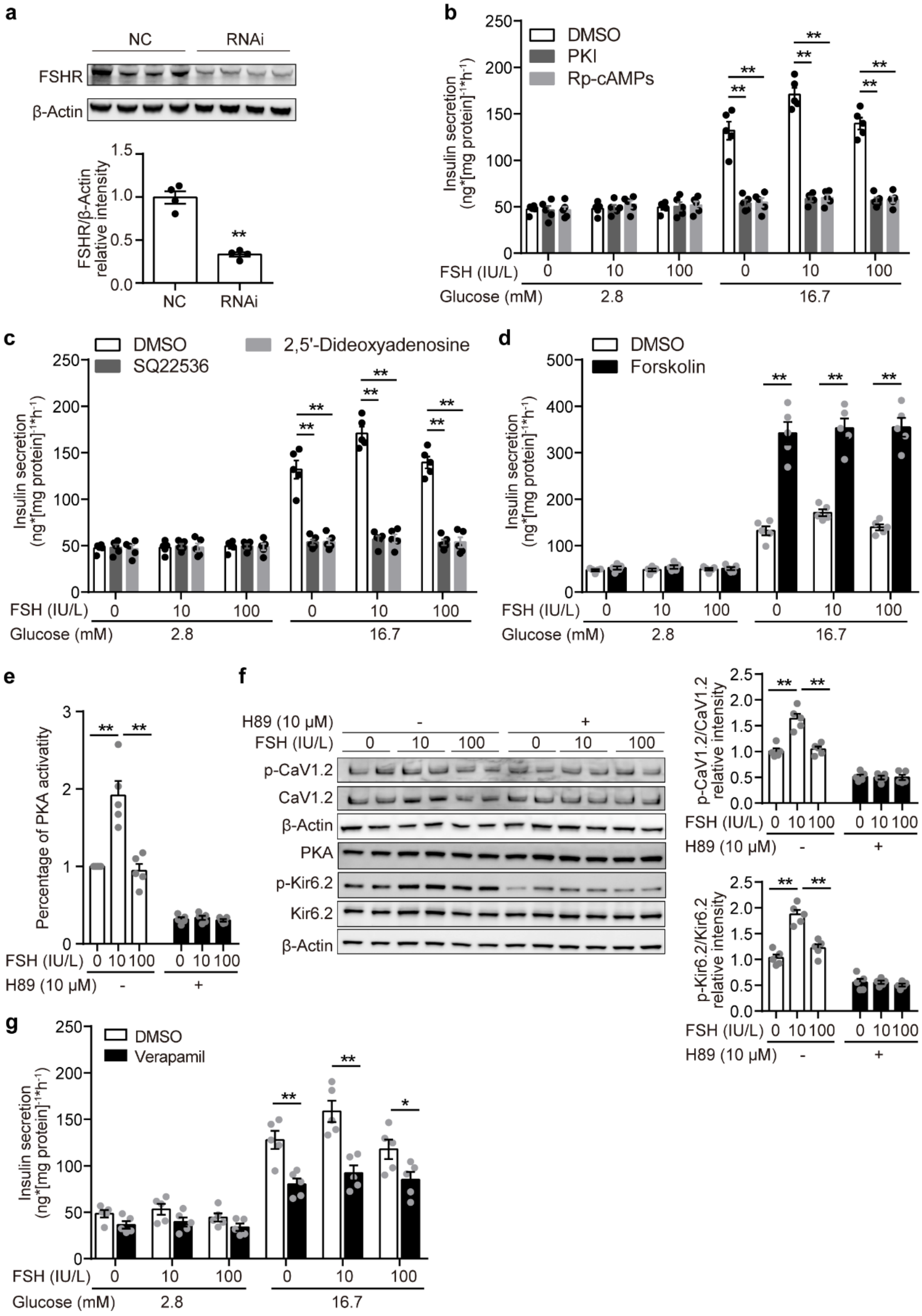

Supplementary Figure 14. The effect of FSH on PKA pathway in MIN6 cells.

(**a**) FSHR protein level in the MIN6 cells transfected with control siRNA (NC) or Fshr siRNA. (**b**) GSIS was tested in MIN6 cells treated with or without PKA inhibitor (PKI,

10 μM; Rp-cAMPs, 10 μM), n = 5 per group, $^{**}P < 0.01$. (**c**) GSIS was tested in MIN6 cells treated with or without AC inhibitor (SQ22536, 10 μM; 2',5'-Dideoxyadenosine, 20 μM), n = 5 per group, $^{**}P < 0.01$. (**d**) GSIS was tested in MIN6 cells treated with or without AC activator (Forskolin, 10 μM), n = 5 per group, $^{**}P < 0.01$. (**e**) PKA activity was measured in MIN6 cells after stimulated with 16.7 mM glucose and different concentrations of FSH for 1 hour with or without PKA inhibitor (H89, 10 μM), n = 5 per group, $^{**}P < 0.01$. (**f**) Representative protein levels of p-CaV1.2, and p-Kir6.2 were analyzed by immunoblotting in the MIN6 cells stimulated with 16.7 mM glucose and different concentrations of FSH for 1 hour with or without PKA inhibitor (H89, 10 μM). (**g**) GSIS was tested in MIN6 cells treated with or without Ca^{2+} channel blocker (verapamil, 10 μM), n = 5 per group, $^{**}P < 0.01$. All the data were shown as mean ± s.e.m. and analyzed by one-way ANOVA (**b, c, e, f**) or unpaired two-tailed Student's *t*-tests (**a, d, g**). Statistical details are in Supplementary Table 2. Source data are provided as a Source Data file.

Supplementary Fig. 15

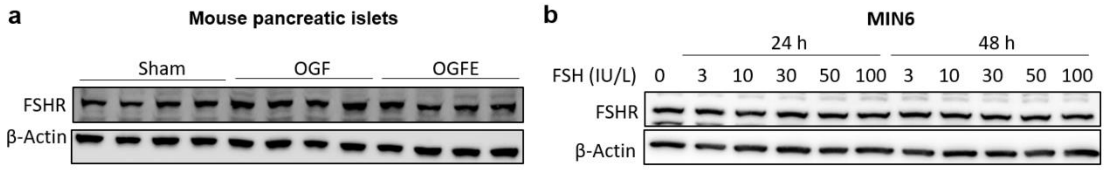

Supplementary Figure 15. High FSH did not affect FSHR expression in the pancreatic islets of the OVX model and MIN6 cells.

(**a**) Western blots were performed to examine FSHR expression in the pancreatic islets of the OVX model. (**b**) MIN6 cells treated with or without FSH for 24 hours or 48 hours, western blots were performed to examine FSHR expression in the MIN6 cells. The experiments were repeated 3 times with similar results.

Supplementary Fig. 16

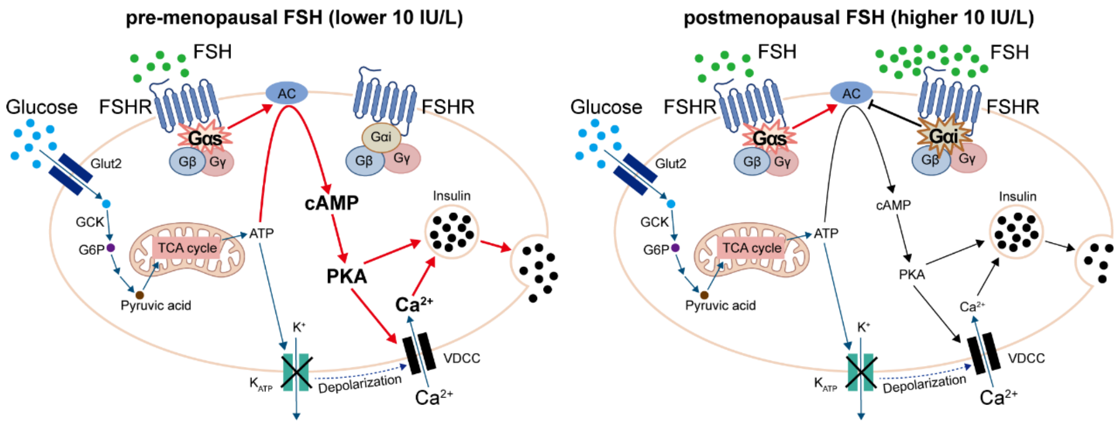

Supplementary Figure 16. Schematic model depicting the proposed role of FSH/FSHR in pancreatic islet β-cells.

Pre-menopausal FSH (lower than 10 IU/L) activates stimulatory Gαs protein via FSHR, leading to adenylate cyclase (AC) activation, promoting the cAMP/PKA pathway and intracellular Ca^{2+} accumulating to enhance GSIS (left). While the FSH level was higher than 10 IU/L, the postmenopausal FSH levels, FSH binding to FSHR could activate Gαi as well, which inhibits the activation of AC and attenuate the cAMP/PKA pathway and intracellular Ca^{2+} signaling, resulting in decreased GSIS (right).

Supplementary Table 1. Primers used for RT-qPCR analysis.

Gene	Forward Primer	Reverse Primer
human FSHR	GTGCGGAACCCCAACATCGTG	ATTCCTTGGATGGGTGTTGTG
human GAPDH	GTGAACCATGAGAAGTATGACAAC	CATGAGTCCTTCCACGATACC
mouse Fshr	GCTACACCCACATCTACCTCACAG	CTGGGCTTGCACCTCATAACAGC
mouse Gapdh	AGGTCGGTGTGAACGGATTTG	TGTAGACCATGTAGTTGAGGTCA
mouse Ins1	CACTTCCTACCCCTGCTGG	ACCACAAAGATGCTGTTTGACA
mouse Ins2	CTTCTTCTACACACCCATGTCCC	CCAAGGTCTGAAGGTCACCTG
mouse β-Actin	AGTGTGACGTTGACATCCGT	GCAGCTCAGTAACAGTCCGC

Supplementary Table 2. Statistical tests.

Figures	Statistical test	Comparisons	*p*-value
2a_FSH	One-way ANOVA with LSD post-test	WT vs. KO	< 0.0001
		WT vs. KO+E2	< 0.0001
2a_E2	One-way ANOVA with LSD post-test	WT vs. KO	< 0.0001
		WT vs. KO+E2	0.7196
2c_GTT and AUC	One-way ANOVA with LSD post-test	0 min: WT vs. KO	0.297
		0 min: WT vs. KO+E2	0.596
		30 min: WT vs. KO	< 0.0001
		30 min: WT vs. KO+E2	< 0.0001
		60 min: WT vs. KO	< 0.0001
		60 min: WT vs. KO+E2	0.002
		120 min: WT vs. KO	0.003
		120 min: WT vs. KO+E2	0.009
		AUC: WT vs. KO	< 0.0001
		AUC: WT vs. KO+E2	< 0.0001
2d_ITT and AUC	One-way ANOVA with LSD post-test	30 min: WT vs. KO	0.184
		30 min: WT vs. KO+E2	0.835
		60 min: WT vs. KO	0.109
		60 min: WT vs. KO+E2	0.283
		90 min: WT vs. KO	0.938
		90 min: WT vs. KO+E2	0.581
		120 min: WT vs. KO	0.615
		120 min: WT vs. KO+E2	0.338
		AUC: WT vs. KO	0.932
		AUC: WT vs. KO+E2	0.986
2e_blood insulin	One-way ANOVA with LSD post-test	0 min: WT vs. KO	0.567
		0 min: WT vs. KO+E2	0.333
		30 min: WT vs. KO	< 0.0001
		30 min: WT vs. KO+E2	< 0.0001
		Fold: WT vs. KO	< 0.0001
		Fold: WT vs. KO+E2	< 0.0001
2f_GTT and AUC	Two-sided student's t-test	0 min: Ctrl vs. CKO	0.317
		30 min: Ctrl vs. CKO	0.003
		60 min: Ctrl vs. CKO	0.02
		120 min: Ctrl vs. CKO	0.007
		AUC: Ctrl vs. CKO	0.004
2g_ITT and AUC	Two-sided student's t-test	30 min: Ctrl vs. CKO	0.971
		60 min: Ctrl vs. CKO	0.32
		90 min: Ctrl vs. CKO	0.844
		120 min: Ctrl vs. CKO	0.622
		AUC: Ctrl vs. CKO	0.599

2h_blood insulin	Two-sided student's t-test	0 min: Ctrl vs. CKO	0.348
		30 min: Ctrl vs. CKO	< 0.0001
		Fold: Ctrl vs. CKO	< 0.0001
3a_GSIS_WT_16.7 mM glucose	One-way ANOVA with LSD post-test	0 IU/L FSH vs. 3 IU/L FSH	< 0.0001
		0 IU/L FSH vs. 10 IU/L FSH	< 0.0001
		0 IU/L FSH vs. 30 IU/L FSH	< 0.0001
		0 IU/L FSH vs. 50 IU/L FSH	0.004
		0 IU/L FSH vs. 100 IU/L FSH	0.053
		3 IU/L FSH vs. 10 IU/L FSH	< 0.0001
		3 IU/L FSH vs. 30 IU/L FSH	0.157
		3 IU/L FSH vs. 50 IU/L FSH	0.019
		3 IU/L FSH vs. 100 IU/L FSH	< 0.0001
		10 IU/L FSH vs. 30 IU/L FSH	0.001
		10 IU/L FSH vs. 50 IU/L FSH	< 0.0001
		10 IU/L FSH vs. 100 IU/L FSH	< 0.0001
		30 IU/L FSH vs. 50 IU/L FSH	< 0.0001
		30 IU/L FSH vs. 100 IU/L FSH	< 0.0001
		50 IU/L FSH vs. 100 IU/L FSH	< 0.0001
3b_dynamic GSIS_WT	One-way ANOVA with LSD post-test (at the indicated time points)	15 min: 0 IU/L FSH vs. 10 IU/L FSH	0.983
		17 min: 0 IU/L FSH vs. 10 IU/L FSH	0.918
		19 min: 0 IU/L FSH vs. 10 IU/L FSH	0.498
		21 min: 0 IU/L FSH vs. 10 IU/L FSH	0.818
		23 min: 0 IU/L FSH vs. 10 IU/L FSH	0.977
		25 min: 0 IU/L FSH vs. 10 IU/L FSH	0.372
		27 min: 0 IU/L FSH vs. 10 IU/L FSH	0.188
		29 min: 0 IU/L FSH vs. 10 IU/L FSH	0.265
		31 min: 0 IU/L FSH vs. 10 IU/L FSH	0.032
		33 min: 0 IU/L FSH vs. 10 IU/L FSH	0.001
		35 min: 0 IU/L FSH vs. 10 IU/L FSH	< 0.0001
		37 min: 0 IU/L FSH vs. 10 IU/L FSH	0.061
		38 min: 0 IU/L FSH vs. 10 IU/L FSH	0.009
		40 min: 0 IU/L FSH vs. 10 IU/L FSH	0.008
		42 min: 0 IU/L FSH vs. 10 IU/L FSH	0.005
		44 min: 0 IU/L FSH vs. 10 IU/L FSH	0.001
		46 min: 0 IU/L FSH vs. 10 IU/L FSH	0.048
		48 min: 0 IU/L FSH vs. 10 IU/L FSH	0.044
		50 min: 0 IU/L FSH vs. 10 IU/L FSH	0.001
		52 min: 0 IU/L FSH vs. 10 IU/L FSH	< 0.0001
		54 min: 0 IU/L FSH vs. 10 IU/L FSH	0.008
		56 min: 0 IU/L FSH vs. 10 IU/L FSH	0.087
		58 min: 0 IU/L FSH vs. 10 IU/L FSH	0.298
		60 min: 0 IU/L FSH vs. 10 IU/L FSH	0.311
		62 min: 0 IU/L FSH vs. 10 IU/L FSH	0.959

		64 min: 0 IU/L FSH vs. 10 IU/L FSH	0.891
		66 min: 0 IU/L FSH vs. 10 IU/L FSH	0.878
		68 min: 0 IU/L FSH vs. 10 IU/L FSH	0.245
		70 min: 0 IU/L FSH vs. 10 IU/L FSH	0.964
		72 min: 0 IU/L FSH vs. 10 IU/L FSH	0.682
		74 min: 0 IU/L FSH vs. 10 IU/L FSH	0.283
		15 min: 10 IU/L FSH vs. 100 IU/L FSH	0.561
		17 min: 10 IU/L FSH vs. 100 IU/L FSH	0.12
		19 min: 10 IU/L FSH vs. 100 IU/L FSH	0.029
		21 min: 10 IU/L FSH vs. 100 IU/L FSH	0.015
		23 min: 10 IU/L FSH vs. 100 IU/L FSH	0.048
		25 min: 10 IU/L FSH vs. 100 IU/L FSH	0.005
		27 min: 10 IU/L FSH vs. 100 IU/L FSH	0.184
		29 min: 10 IU/L FSH vs. 100 IU/L FSH	0.001
		31 min: 10 IU/L FSH vs. 100 IU/L FSH	0.007
		33 min: 10 IU/L FSH vs. 100 IU/L FSH	< 0.0001
		35 min: 10 IU/L FSH vs. 100 IU/L FSH	< 0.0001
		37 min: 10 IU/L FSH vs. 100 IU/L FSH	< 0.0001
		38 min: 10 IU/L FSH vs. 100 IU/L FSH	< 0.0001
		40 min: 10 IU/L FSH vs. 100 IU/L FSH	< 0.0001
		42 min: 10 IU/L FSH vs. 100 IU/L FSH	0.001
		44 min: 10 IU/L FSH vs. 100 IU/L FSH	< 0.0001
		46 min: 10 IU/L FSH vs. 100 IU/L FSH	0.005
		48 min: 10 IU/L FSH vs. 100 IU/L FSH	0.011

		50 min: 10 IU/L FSH vs. 100 IU/L FSH	< 0.0001
		52 min: 10 IU/L FSH vs. 100 IU/L FSH	< 0.0001
		54 min: 10 IU/L FSH vs. 100 IU/L FSH	0.004
		56 min: 10 IU/L FSH vs. 100 IU/L FSH	0.006
		58 min: 10 IU/L FSH vs. 100 IU/L FSH	0.164
		60 min: 10 IU/L FSH vs. 100 IU/L FSH	0.465
		62 min: 10 IU/L FSH vs. 100 IU/L FSH	0.191
		64 min: 10 IU/L FSH vs. 100 IU/L FSH	0.134
		66 min: 10 IU/L FSH vs. 100 IU/L FSH	0.055
		68 min: 10 IU/L FSH vs. 100 IU/L FSH	0.011
		70 min: 10 IU/L FSH vs. 100 IU/L FSH	0.033
		72 min: 10 IU/L FSH vs. 100 IU/L FSH	0.011
		74 min: 10 IU/L FSH vs. 100 IU/L FSH	0.077
4b_FSH	One-way ANOVA with LSD post-test	Sham vs. OGF	< 0.0001
		Sham vs. OGFE	< 0.0001
		OGF vs. OGFE	0.4
4b_E2	One-way ANOVA with LSD post-test	Sham vs. OGF	< 0.0001
		Sham vs. OGFE	0.798
		OGF vs. OGFE	< 0.0001
4c_GTT and AUC	One-way ANOVA with LSD post-test	0 min: Sham vs. OGF	0.81
		0 min: Sham vs. OGFE	0.62
		0 min: OGF vs. OGFE	0.461
		30 min: Sham vs. OGF	< 0.0001
		30 min: Sham vs. OGFE	0.007
		30 min: OGF vs. OGFE	0.003
		60 min: Sham vs. OGF	< 0.0001
		60 min: Sham vs. OGFE	0.002
		60 min: OGF vs. OGFE	0.089
		120 min: Sham vs. OGF	0.009
		120 min: Sham vs. OGFE	0.167

		120 min: OGF vs. OGFE	0.21
		AUC: Sham vs. OGF	< 0.0001
		AUC: Sham vs. OGFE	0.005
		AUC: OGF vs. OGFE	0.027
4d_ITT and AUC	One-way ANOVA with LSD post-test	30 min: Sham vs. OGF	0.133
		30 min: Sham vs. OGFE	0.944
		30 min: OGF vs. OGFE	0.167
		60 min: Sham vs. OGF	0.06
		60 min: Sham vs. OGFE	0.32
		60 min: OGF vs. OGFE	0.497
		90 min: Sham vs. OGF	0.886
		90 min: Sham vs. OGFE	0.624
		90 min: OGF vs. OGFE	0.532
		120 min: Sham vs. OGF	0.831
		120 min: Sham vs. OGFE	0.368
		120 min: OGF vs. OGFE	0.483
		AUC: Sham vs. OGF	0.151
		AUC: Sham vs. OGFE	0.84
		AUC: OGF vs. OGFE	0.232
4e_blood insulin	One-way ANOVA with LSD post-test	0 min: Sham vs. OGF	0.759
		0 min: Sham vs. OGFE	0.869
		0 min: OGF vs. OGFE	0.878
		30 min: Sham vs. OGF	< 0.0001
		30 min: Sham vs. OGFE	< 0.0001
		30 min: OGF vs. OGFE	0.058
		Fold: Sham vs. OGF	< 0.0001
		Fold: Sham vs. OGFE	< 0.0001
		Fold: OGF vs. OGFE	0.008
5a_GSIS_NC_16.7 mM glucose	One-way ANOVA with LSD post-test	0 IU/L FSH vs. 3 IU/L FSH	< 0.0001
		0 IU/L FSH vs. 10 IU/L FSH	< 0.0001
		0 IU/L FSH vs. 30 IU/L FSH	0.003
		0 IU/L FSH vs. 50 IU/L FSH	0.125
		0 IU/L FSH vs. 100 IU/L FSH	0.405
		3 IU/L FSH vs. 10 IU/L FSH	0.005
		3 IU/L FSH vs. 30 IU/L FSH	0.392
		3 IU/L FSH vs. 50 IU/L FSH	0.017
		3 IU/L FSH vs. 100 IU/L FSH	< 0.0001
		10 IU/L FSH vs. 30 IU/L FSH	< 0.0001
		10 IU/L FSH vs. 50 IU/L FSH	< 0.0001
		10 IU/L FSH vs. 100 IU/L FSH	< 0.0001
		30 IU/L FSH vs. 50 IU/L FSH	0.115
		30 IU/L FSH vs. 100 IU/L FSH	< 0.0001
		50 IU/L FSH vs. 100 IU/L FSH	0.02

5b_cAMP	One-way ANOVA with LSD post-test	0 IU/L FSH vs. 3 IU/L FSH	< 0.0001
		0 IU/L FSH vs. 10 IU/L FSH	< 0.0001
		0 IU/L FSH vs. 30 IU/L FSH	< 0.0001
		0 IU/L FSH vs. 50 IU/L FSH	< 0.0001
		0 IU/L FSH vs. 100 IU/L FSH	0.63
		3 IU/L FSH vs. 10 IU/L FSH	< 0.0001
		3 IU/L FSH vs. 30 IU/L FSH	0.397
		3 IU/L FSH vs. 50 IU/L FSH	0.667
		3 IU/L FSH vs. 100 IU/L FSH	< 0.0001
		10 IU/L FSH vs. 30 IU/L FSH	0.004
		10 IU/L FSH vs. 50 IU/L FSH	< 0.0001
		10 IU/L FSH vs. 100 IU/L FSH	< 0.0001
		30 IU/L FSH vs. 50 IU/L FSH	0.204
		30 IU/L FSH vs. 100 IU/L FSH	< 0.0001
		50 IU/L FSH vs. 100 IU/L FSH	< 0.0001
5c_PKA	One-way ANOVA with LSD post-test	0 IU/L FSH vs. 3 IU/L FSH	< 0.0001
		0 IU/L FSH vs. 10 IU/L FSH	< 0.0001
		0 IU/L FSH vs. 30 IU/L FSH	< 0.0001
		0 IU/L FSH vs. 50 IU/L FSH	< 0.0001
		0 IU/L FSH vs. 100 IU/L FSH	0.578
		3 IU/L FSH vs. 10 IU/L FSH	< 0.0001
		3 IU/L FSH vs. 30 IU/L FSH	0.014
		3 IU/L FSH vs. 50 IU/L FSH	0.649
		3 IU/L FSH vs. 100 IU/L FSH	< 0.0001
		10 IU/L FSH vs. 30 IU/L FSH	0.001
		10 IU/L FSH vs. 50 IU/L FSH	< 0.0001
		10 IU/L FSH vs. 100 IU/L FSH	< 0.0001
		30 IU/L FSH vs. 50 IU/L FSH	0.004
		30 IU/L FSH vs. 100 IU/L FSH	< 0.0001
		50 IU/L FSH vs. 100 IU/L FSH	< 0.0001
5d_GSIS_16.7 mM glucose	One-way ANOVA with LSD post-test	0 IU/L FSH: DMSO vs. H89	< 0.0001
		10 IU/L FSH: DMSO vs. H89	< 0.0001
		100 IU/L FSH: DMSO vs. H89	< 0.0001
		DMSO: 0 IU/L FSH vs. 10 IU/L FSH	< 0.0001
		DMSO: 10 IU/L FSH vs. 100 IU/L FSH	0.002
5e_Ca^{2+} levels	One-way ANOVA with LSD post-test (at the indicated time points)	0 min: 0 IU/L FSH vs. 10 IU/L FSH	0.535
		5 min: 0 IU/L FSH vs. 10 IU/L FSH	0.316
		10 min: 0 IU/L FSH vs. 10 IU/L FSH	0.164
		15 min: 0 IU/L FSH vs. 10 IU/L FSH	0.005
		20 min: 0 IU/L FSH vs. 10 IU/L FSH	0.001
		25 min: 0 IU/L FSH vs. 10 IU/L FSH	0.003
		30 min: 0 IU/L FSH vs. 10 IU/L FSH	0.007

		35 min: 0 IU/L FSH vs. 10 IU/L FSH	0.004
		40 min: 0 IU/L FSH vs. 10 IU/L FSH	0.017
		45 min: 0 IU/L FSH vs. 10 IU/L FSH	0.011
		50 min: 0 IU/L FSH vs. 10 IU/L FSH	0.006
		55 min: 0 IU/L FSH vs. 10 IU/L FSH	0.009
		60 min: 0 IU/L FSH vs. 10 IU/L FSH	0.071
		0 min: 10 IU/L FSH vs. 100 IU/L FSH	0.539
		5 min: 10 IU/L FSH vs. 100 IU/L FSH	0.465
		10 min: 10 IU/L FSH vs. 100 IU/L FSH	0.07
		15 min: 10 IU/L FSH vs. 100 IU/L FSH	0.011
		20 min: 10 IU/L FSH vs. 100 IU/L FSH	0.002
		25 min: 10 IU/L FSH vs. 100 IU/L FSH	0.005
		30 min: 10 IU/L FSH vs. 100 IU/L FSH	0.008
		35 min: 10 IU/L FSH vs. 100 IU/L FSH	0.008
		40 min: 10 IU/L FSH vs. 100 IU/L FSH	0.006
		45 min: 10 IU/L FSH vs. 100 IU/L FSH	0.009
		50 min: 10 IU/L FSH vs. 100 IU/L FSH	0.002
		55 min: 10 IU/L FSH vs. 100 IU/L FSH	0.009
		60 min: 10 IU/L FSH vs. 100 IU/L FSH	0.114
5g_GSIS and cAMP	Two-sided student’s t-test	2.8 mM glucose: DMSO vs. NF449	0.063
		16.7 mM glucose: DMSO vs. NF449	0.039
		cAMP: DMSO vs. NF449	0.002
5h_GSIS_16.7 mM glucose and cAMP	Two-sided student’s t-test	2.8 mM glucose: DMSO vs. NF449	0.499
		16.7 mM glucose: DMSO vs. NF449	0.049
		cAMP: DMSO vs. NF449	0.031
5k_GSIS_16.7 mM glucose and cAMP	Two-sided student’s t-test	2.8 mM glucose: DMSO vs. PTX	0.476
		16.7 mM glucose: DMSO vs. PTX	0.006
		cAMP: DMSO vs. PTX	0.01
Supplementary Fig. 3b_GSIS	Two-sided student’s t-test	16.7 mM: WT vs. KO	0.010202

Supplementary Fig. 4b_GTT and AUC	Two-sided student's t-test	0 min: WT vs. KO	0.008
		30 min: WT vs. KO	0.019
		60 min: WT vs. KO	0.151
		120 min: WT vs. KO	0.603
		AUC: WT vs. KO	0.025
Supplementary Fig. 4d_blood insulin	Two-sided student's t-test	0 min: WT vs. KO	0.907
		30 min: WT vs. KO	< 0.0001
		Fold: WT vs. KO	< 0.0001
Supplementary Fig. 4f_GTT and AUC	Two-sided student's t-test	0 min: Ctrl vs. CKO	0.148
		30 min: Ctrl vs. CKO	0.012
		60 min: Ctrl vs. CKO	0.031
		120 min: Ctrl vs. CKO	0.172
		AUC: Ctrl vs. CKO	0.029
Supplementary Fig. 4h_blood insulin	Two-sided student's t-test	0 min: Ctrl vs. CKO	0.416
		30 min: Ctrl vs. CKO	< 0.0001
		Fold: Ctrl vs. CKO	< 0.0001
Supplementary Fig. 5	One-way ANOVA with LSD post-test	for all	>0.05
Supplementary Fig. 6_GSIS_WT_16.7 mM glucose	One-way ANOVA with LSD post-test	0 IU/L FSH vs. 3 IU/L FSH	0.001
		0 IU/L FSH vs. 10 IU/L FSH	< 0.0001
		0 IU/L FSH vs. 30 IU/L FSH	0.354
		0 IU/L FSH vs. 50 IU/L FSH	0.788
		0 IU/L FSH vs. 100 IU/L FSH	0.544
		3 IU/L FSH vs. 10 IU/L FSH	0.812
		3 IU/L FSH vs. 30 IU/L FSH	0.007
		3 IU/L FSH vs. 50 IU/L FSH	< 0.0001
		3 IU/L FSH vs. 100 IU/L FSH	< 0.0001
		10 IU/L FSH vs. 30 IU/L FSH	0.004
		10 IU/L FSH vs. 50 IU/L FSH	< 0.0001
		10 IU/L FSH vs. 100 IU/L FSH	< 0.0001
		30 IU/L FSH vs. 50 IU/L FSH	0.235
		30 IU/L FSH vs. 100 IU/L FSH	0.132
		50 IU/L FSH vs. 100 IU/L FSH	0.735
Supplementary Fig. 7a_GSIS_16.7 mM glucose	One-way ANOVA with LSD post-test	0 IU/FSH: NC vs. FSHR OVE	0.826
		10 IU/FSH: NC vs. FSHR OVE	0.961
		100 IU/FSH: NC vs. FSHR OVE	0.873
Supplementary Fig. 7b_GSIS_16.7 mM glucose	One-way ANOVA with LSD post-test	0 IU/FSH: NC vs. FSHR OVE	0.767
		10 IU/FSH: NC vs. FSHR OVE	0.92
		100 IU/FSH: NC vs. FSHR OVE	0.709
Supplementary Fig. 9a	One-way ANOVA with LSD post-test	for all	>0.05
Supplementary Fig. 9b	One-way ANOVA with LSD post-test	for all	>0.05

Supplementary Fig. 10a_body weight	One-way ANOVA with LSD post-test	8w: Sham vs. OGF	0.977
		8w: Sham vs. OGFE	0.964
		8w: OGF vs. OGFE	0.945
		9w: Sham vs. OGF	0.017
		9w: Sham vs. OGFE	0.053
		9w: OGF vs. OGFE	0.618
		10w: Sham vs. OGF	< 0.0001
		10w: Sham vs. OGFE	< 0.0001
		10w: OGF vs. OGFE	0.972
		11w: Sham vs. OGF	< 0.0001
		11w: Sham vs. OGFE	< 0.0001
		11w: OGF vs. OGFE	0.546
		12w: Sham vs. OGF	< 0.0001
		12w: Sham vs. OGFE	< 0.0001
		12w: OGF vs. OGFE	0.202
		13w: Sham vs. OGF	< 0.0001
		13w: Sham vs. OGFE	< 0.0001
		13w: OGF vs. OGFE	0.377
Supplementary Fig. 11a_GTT and AUC	Two-sided student's t-test	0 min: WT vs. KO	0.164
		30 min: WT vs. KO	0.011
		60 min: WT vs. KO	< 0.0001
		120 min: WT vs. KO	0.001
		AUC: WT vs. KO	< 0.0001
Supplementary Fig. 11b_GTT and AUC	Two-sided student's t-test	0 min: WT vs. KO	0.075
		30 min: WT vs. KO	0.001
		60 min: WT vs. KO	0.003
		120 min: WT vs. KO	0.208
		AUC: WT vs. KO	0.003
Supplementary Fig. 12a_GTT and AUC	Two-sided student's t-test	Ctrl vs. HFD	0.801
		WT vs. db/db	0.836
Supplementary Fig. 13	One-way ANOVA with LSD post-test	for all	>0.05
Supplementary Fig. 14a	Two-sided student's t-test	NC vs. RNAi	< 0.0001
Supplementary Fig. 14b_GSIS_16.7 mM glucose	One-way ANOVA with LSD post-test	0 IU/L FSH: DMSO vs. PKI	< 0.0001
		0 IU/L FSH: DMSO vs. Rp-cAMPs	< 0.0001
		10 IU/L FSH: DMSO vs. PKI	< 0.0001
		10 IU/L FSH: DMSO vs. Rp-cAMPs	< 0.0001
		100 IU/L FSH: DMSO vs. PKI	< 0.0001
		100 IU/L FSH: DMSO vs. Rp-cAMPs	< 0.0001
Supplementary Fig. 14c_GSIS_16.7 mM glucose	One-way ANOVA with LSD post-test	0 IU/L FSH: DMSO vs. SQ22536	< 0.0001
		0 IU/L FSH: DMSO vs. 2',5'-Dideoxyadenosine	< 0.0001

		10 IU/L FSH: DMSO vs. SQ22536	< 0.0001
		10 IU/L FSH: DMSO vs. 2',5'-Dideoxyadenosine	< 0.0001
		100 IU/L FSH: DMSO vs. SQ22536	< 0.0001
		100 IU/L FSH: DMSO vs. 2',5'-Dideoxyadenosine	< 0.0001
Supplementary Fig. 14d_GSIS_16.7 mM glucose	One-way ANOVA with LSD post-test	0 IU/L FSH: DMSO vs. Forskolin	< 0.0001
		10 IU/L FSH: DMSO vs. Forskolin	< 0.0001
		100 IU/L FSH: DMSO vs. Forskolin	< 0.0001
Supplementary Fig. 14e	One-way ANOVA with LSD post-test	H89-: 0 IU/L FSH vs.10 IU/L FSH	< 0.0001
		H89-: 10 IU/L FSH vs.100 IU/L FSH	< 0.0001
		H89+: 0 IU/L FSH vs.10 IU/L FSH	0.88
		H89+: 10 IU/L FSH vs.100 IU/L FSH	0.787
Supplementary Fig. 14f_pCaV1.2/CaV1.2	One-way ANOVA with LSD post-test	H89-: 0 IU/L FSH vs.10 IU/L FSH	< 0.0001
		H89-: 10 IU/L FSH vs.100 IU/L FSH	< 0.0001
		H89+: 0 IU/L FSH vs.10 IU/L FSH	0.807
		H89+: 10 IU/L FSH vs.100 IU/L FSH	0.98
Supplementary Fig. 14f_pKir6.2/Kir6.2	One-way ANOVA with LSD post-test	H89-: 0 IU/L FSH vs.10 IU/L FSH	< 0.0001
		H89-: 10 IU/L FSH vs.100 IU/L FSH	< 0.0001
		H89+: 0 IU/L FSH vs.10 IU/L FSH	0.991
		H89+: 10 IU/L FSH vs.100 IU/L FSH	0.547
Supplementary Fig. 14g_GSIS_16.7 mM glucose	One-way ANOVA with LSD post-test	0 IU/L FSH: DMSO vs. Verapamil	0.001
		10 IU/L FSH: DMSO vs. Verapamil	< 0.0001
		100 IU/L FSH: DMSO vs. Verapamil	0.019

Source Data of Western blot in Supplementary Figures.

Related to supplementary Fig. 1b

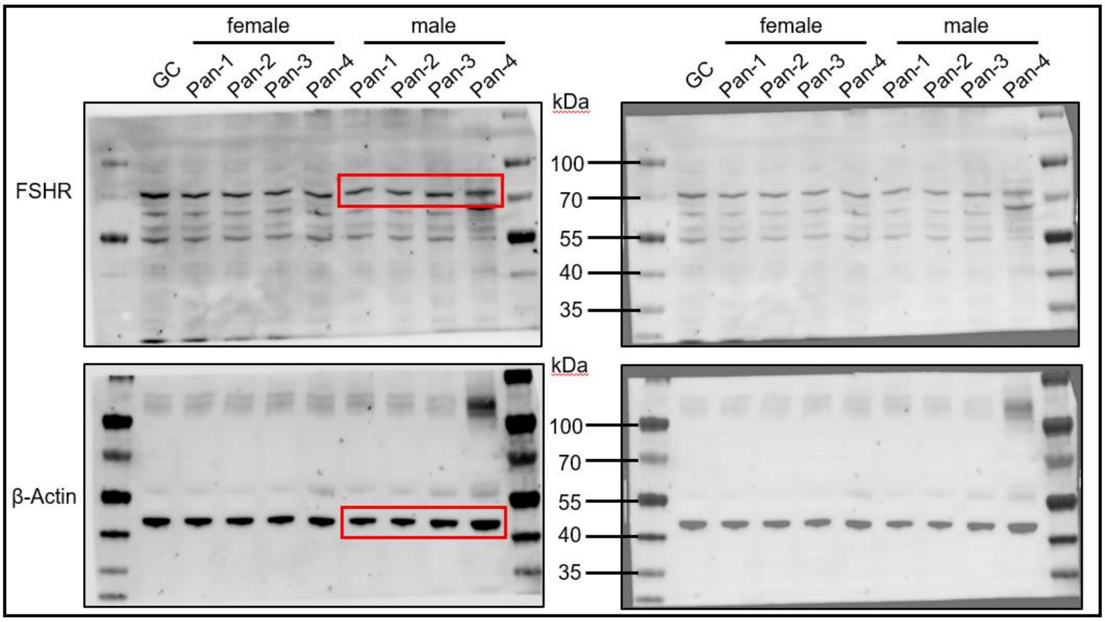

Related to supplementary Fig. 2

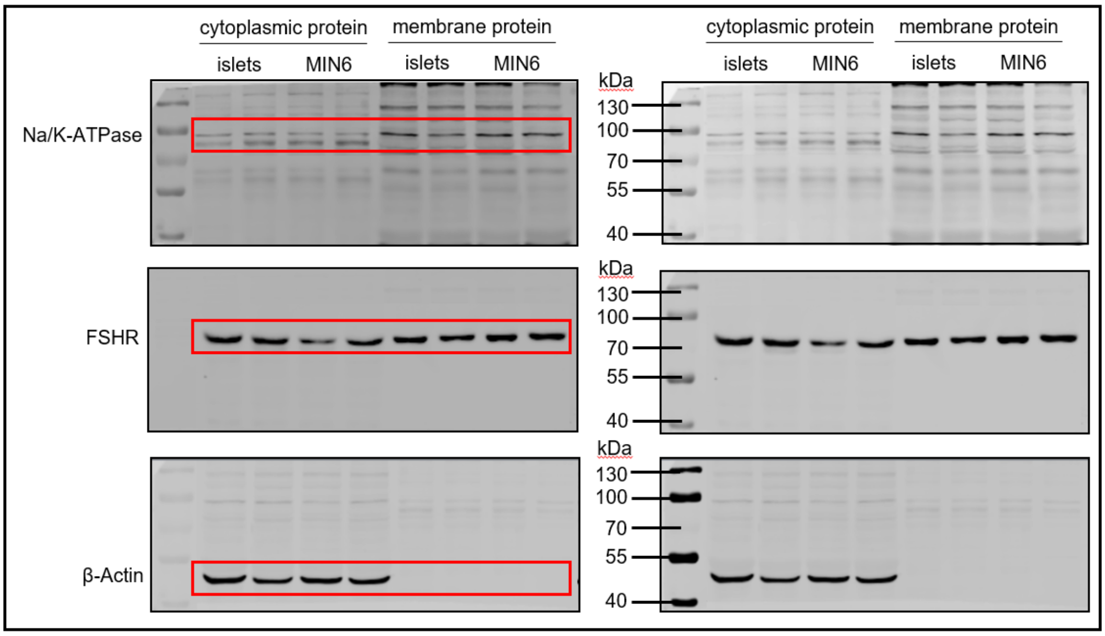

Related to supplementary Fig. 8a

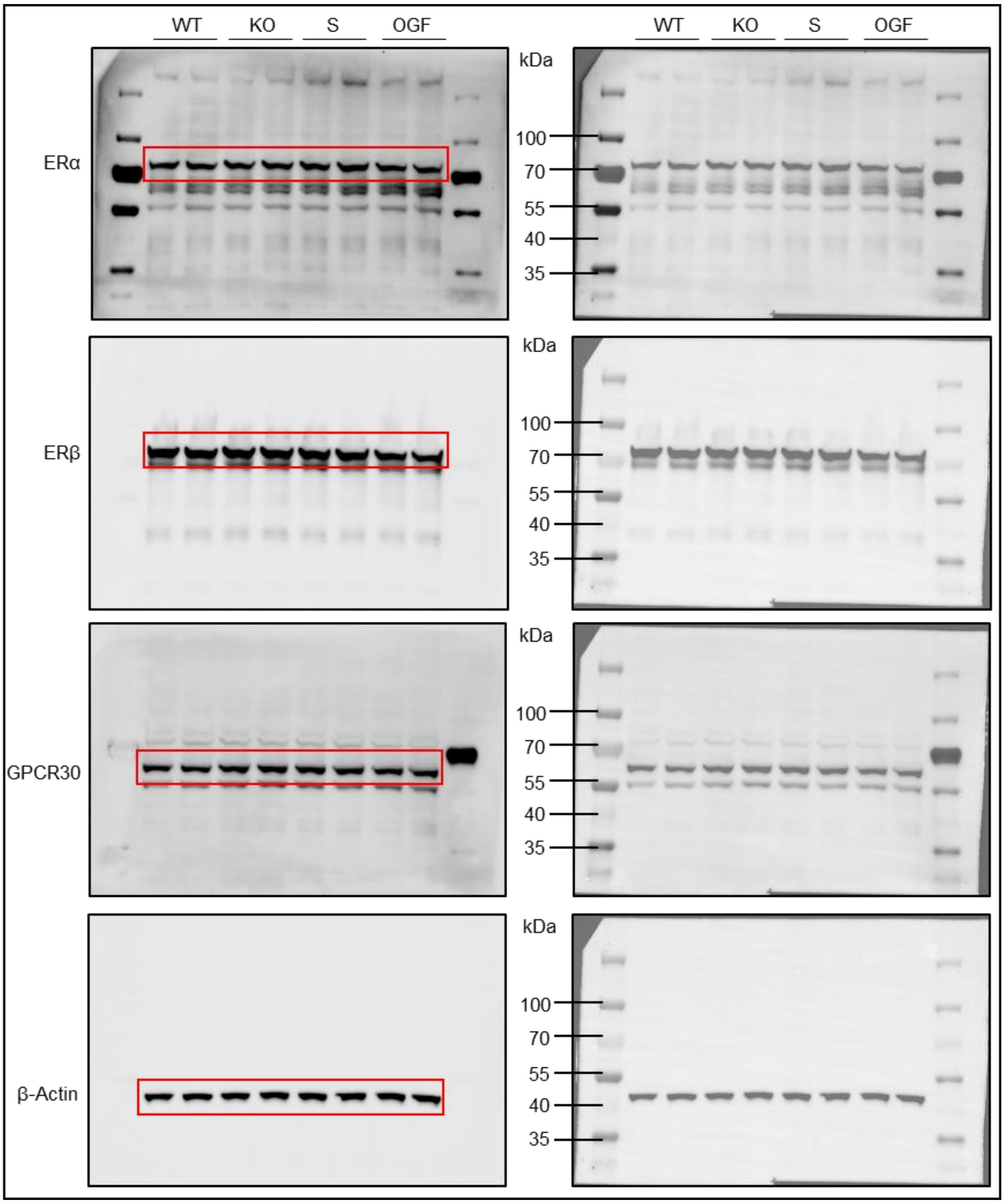

184 Related to supplementary Fig. 8b

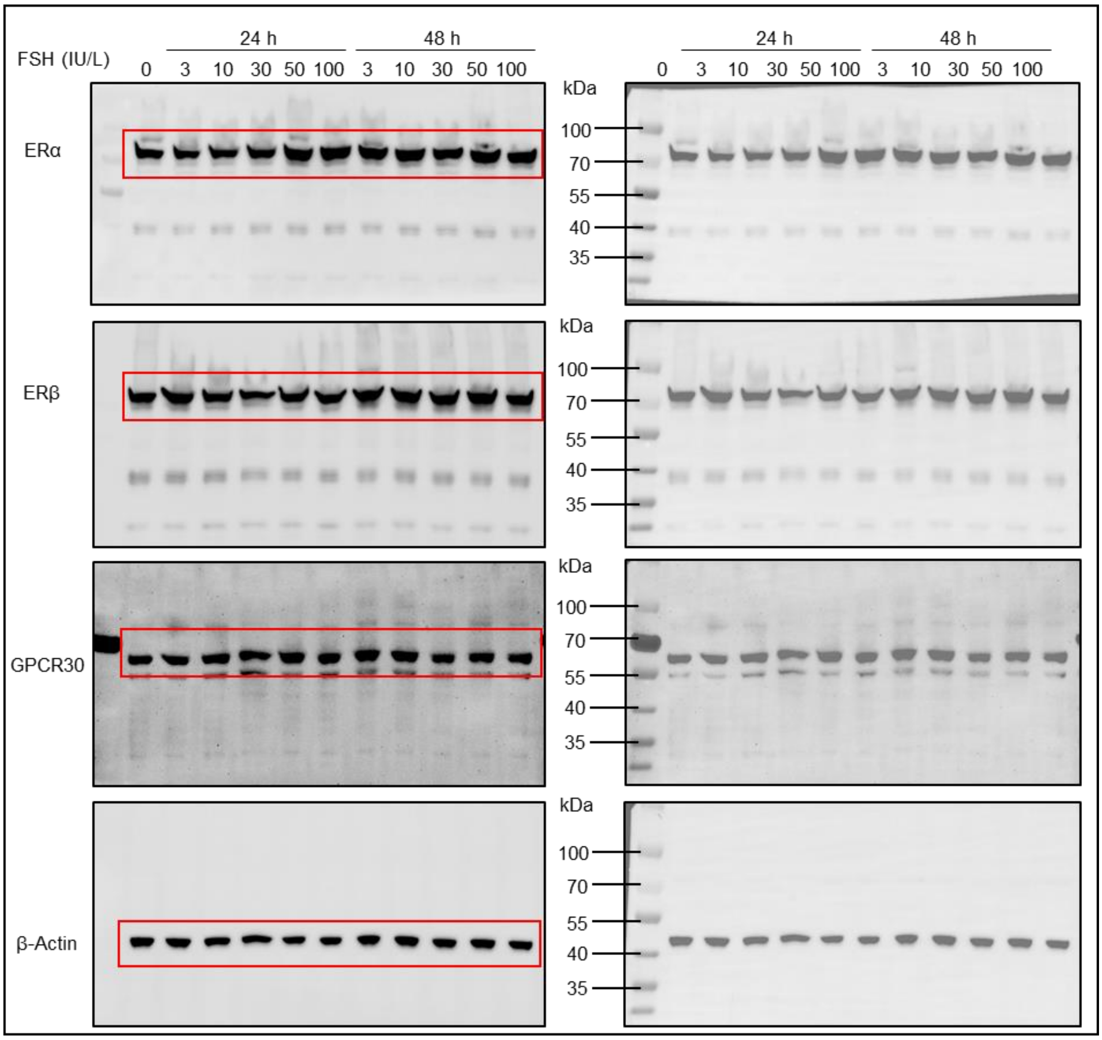

185

186 Related to supplementary Fig. 12b

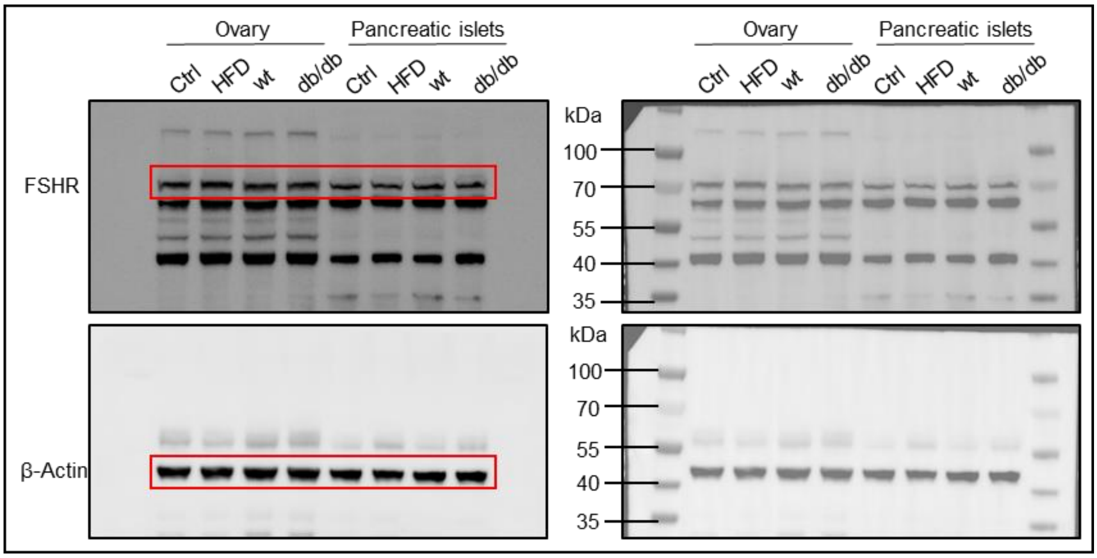

187

Related to supplementary Fig. 14a

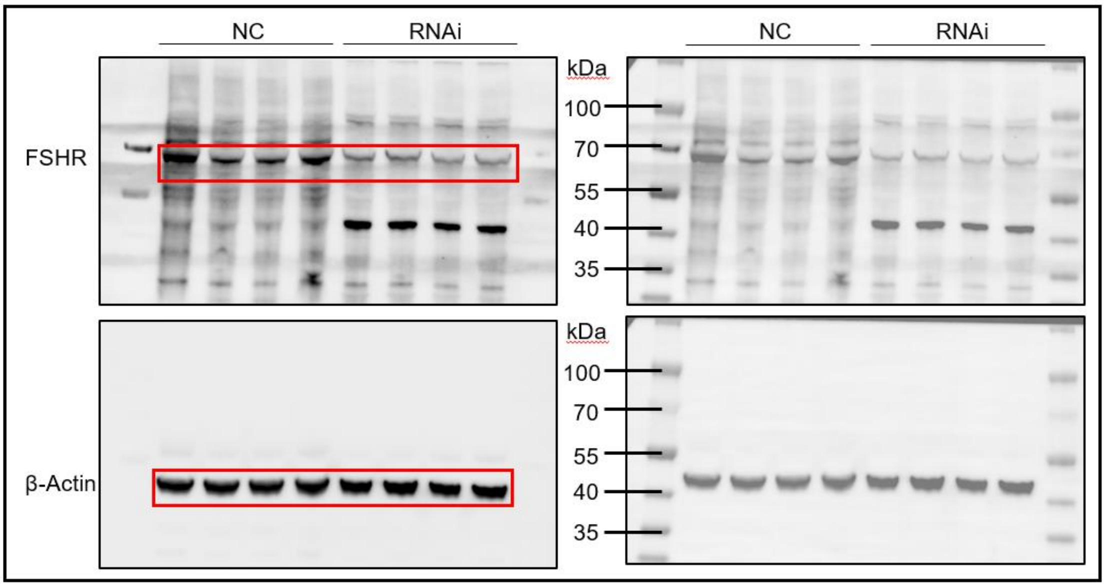

Related to supplementary Fig. 14f

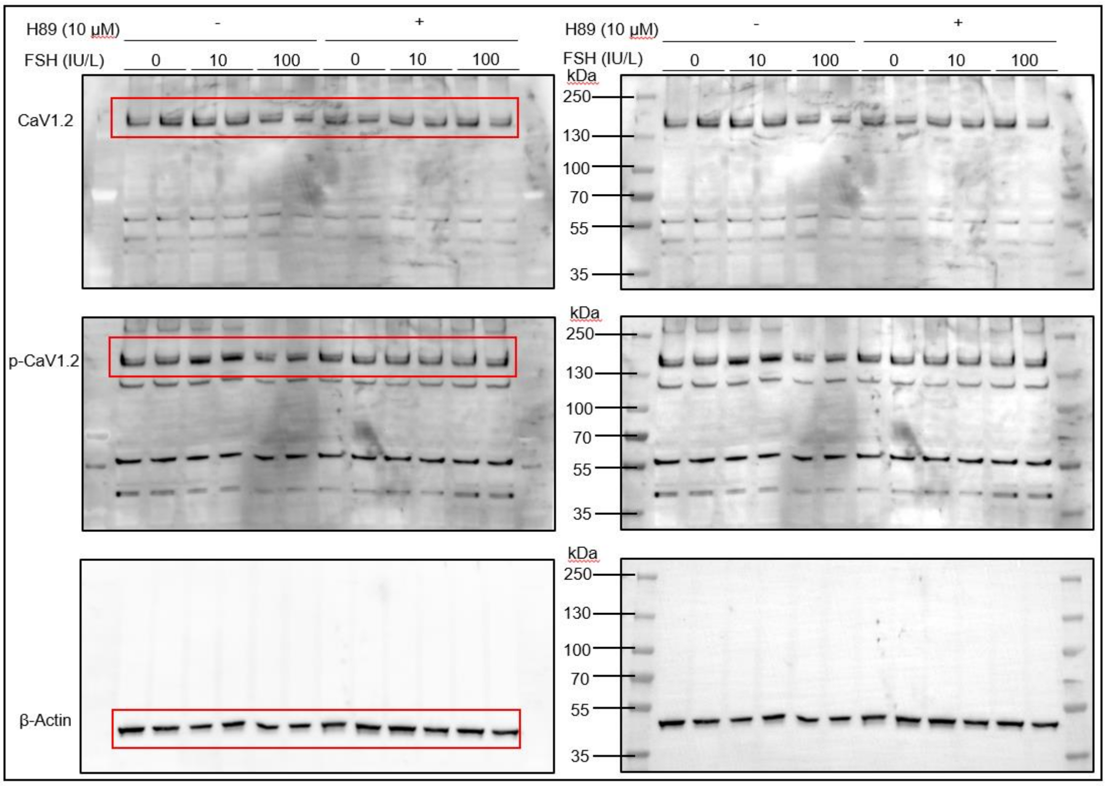

192 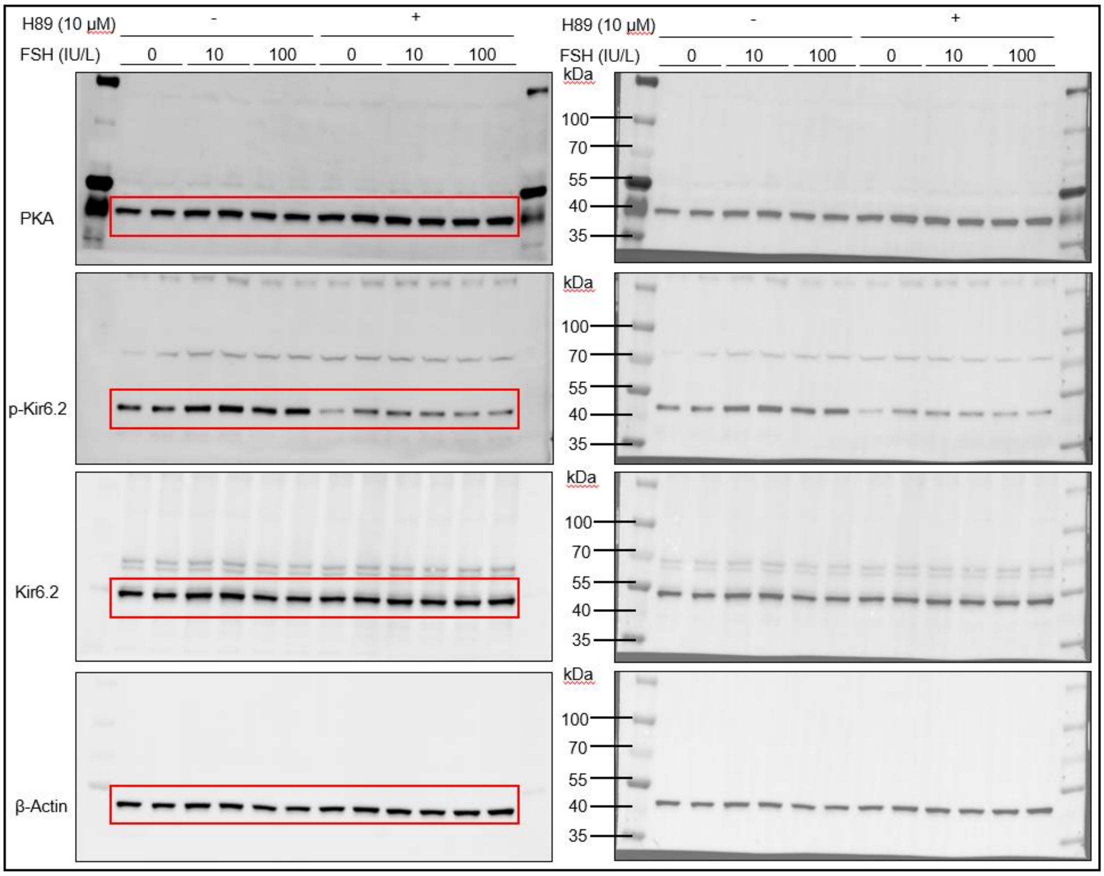
H89 (10 μM)
-
+
FSH (IU/L)
0
10
100
0
10
100
PKA
p-Kir6.2
Kir6.2
β-Actin
kDa
100
70
55
40
35

Related to supplementary Fig. 15a

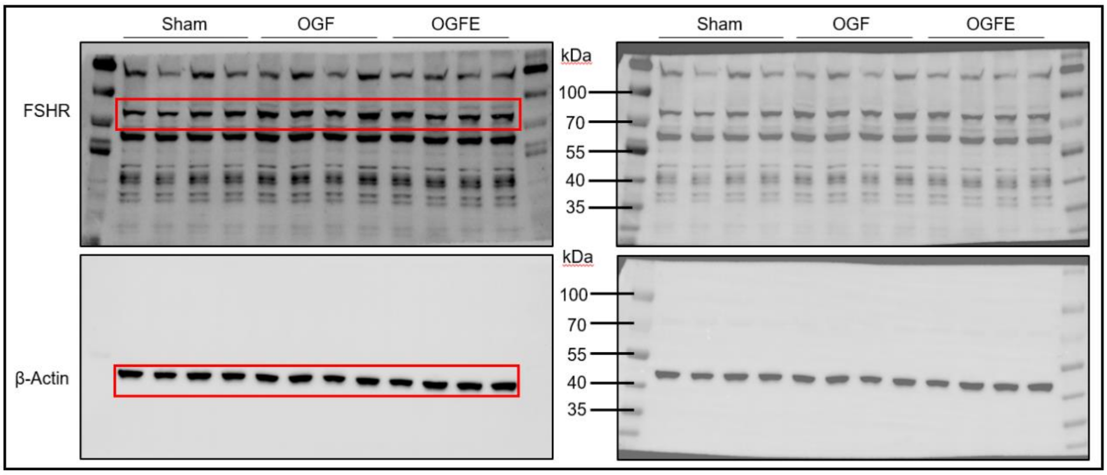

Related to supplementary Fig. 15b

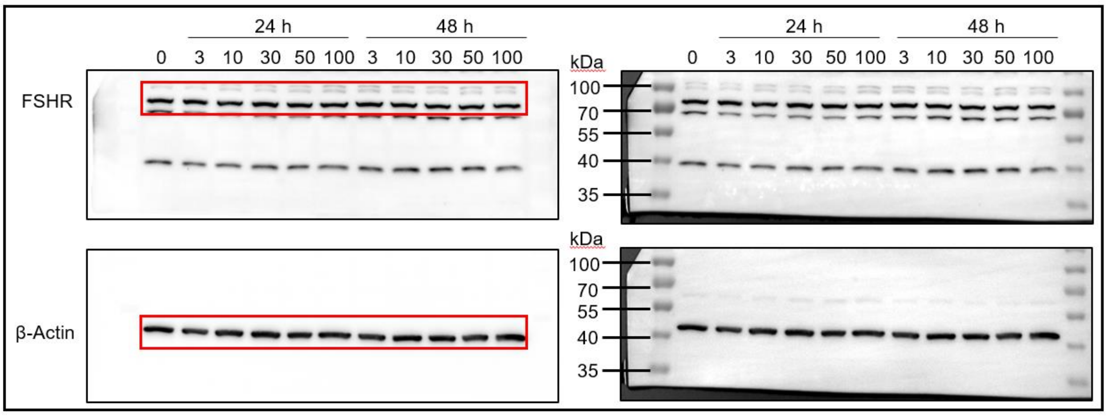

Supplement: Supplementary file 1 — Supplementary Information [file 41467_2023_42801_MOESM1_ESM.pdf]
