# Supplementary figures and images for: Follicle-stimulating hormone orchestrates glucose-stimulated insulin secretion of pancreatic islets
Source: Nat Commun. 2023 Nov 1;14:6991. doi: 10.1038/s41467-023-42801-6 (PMC10620214; doi:10.1038/s41467-023-42801-6)

1 **Figure 1. FSHR expression in human and mouse pancreatic  $\beta$ -cells and MIN6 cells.**

2 Fig 1c.

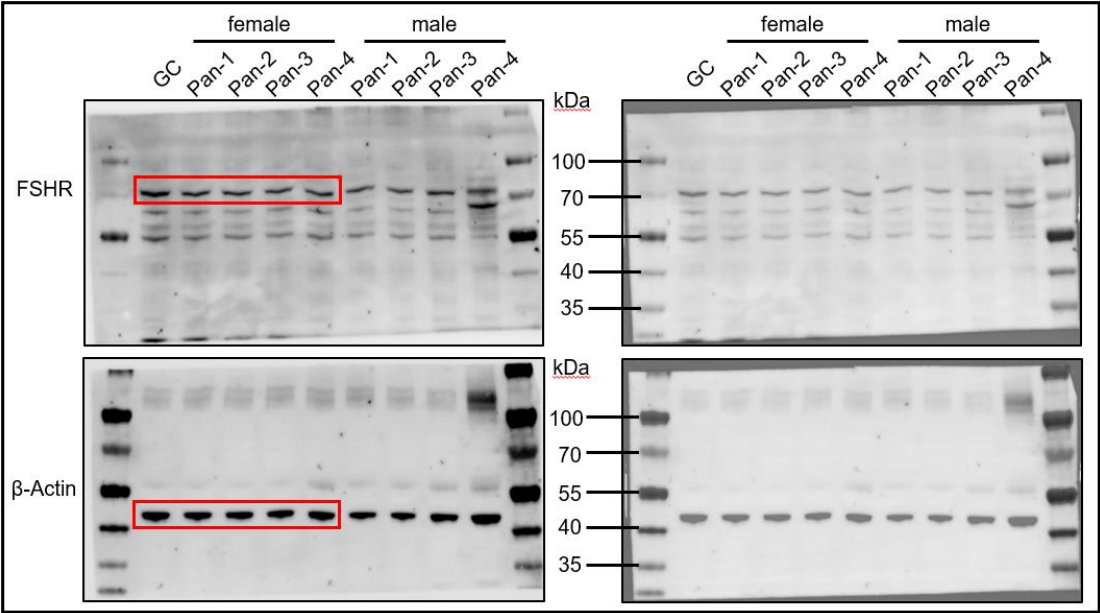

3

4 Fig 1d.

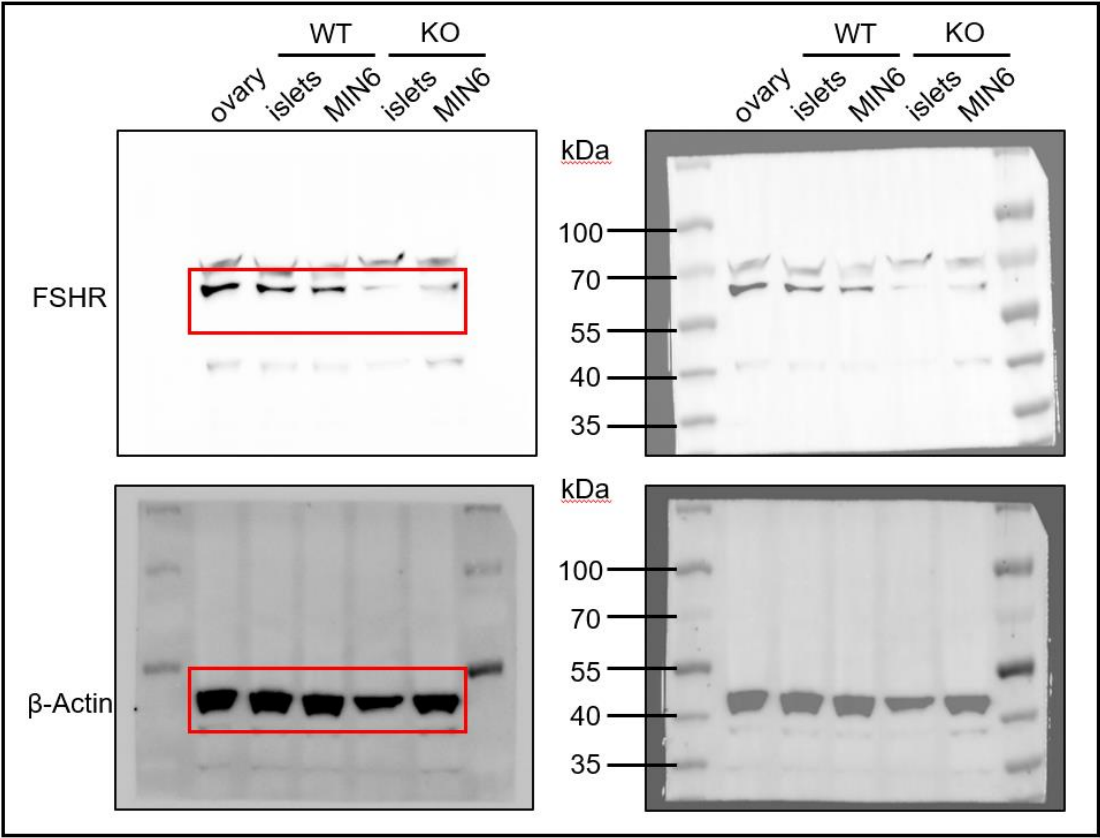

5

Supplement: Supplementary file 3 — Source Data [file 41467_2023_42801_MOESM3_ESM.zip › Source Data of western blot.pdf]
